# *Bawei Chenxiang Wan* ameliorates right ventricular hypertrophy in rats with high altitude heart disease by SIRT3-HIF1α-PDK/PDH signaling pathway improving fatty acid and glucose metabolism

**DOI:** 10.1186/s12906-024-04490-6

**Published:** 2024-05-15

**Authors:** Yiwei Han, Shadi Li, Zhiying Zhang, Xin Ning, Jiajia Wu, Xiaoying Zhang

**Affiliations:** 1 School of Medicine, Xizang Minzu University, Wenhui Road East, Weicheng District, Xianyang, Shaanxi 712082 P.R. China; 2 Engineering Research Center of Tibetan Medicine Detection Technology, Ministry of Education, Xianyang, Shaanxi 712082 P.R. China; 3 Joint Laboratory for Research On Active Components and Pharmacological Mechanism of Tibetan Materia Medica of Tibetan Medical Research Center of Tibet, Xianyang, Shaanxi 712082 P.R. China

**Keywords:** Right ventricular hypertrophy, *Bawei Chenxiang Wan*, SIRT3, HIF1α, PDK, PDH

## Abstract

**Background:**

*Bawei Chenxiang Wan* (*BCW*) is among the most effective and widely used therapies for coronary heart disease and angina pectoris in Tibet. However, whether it confers protection through a right-ventricle (RV) myocardial metabolic mechanism is unknown.

**Methods:**

Male Sprague–Dawley rats were orally administrated with *BCW*, which was injected concurrently with a bolus of Sugen5416, and subjected to hypoxia exposure (SuHx; 5000 m altitude) for 4 weeks. Right ventricular hypertrophy (RVH) in high-altitude heart disease (HAHD) was assessed using Fulton’s index (FI; ratio of RV to left ventricle + septum weights) and heart-weight-to-body-weight ratio (HW/BW). The effect of therapeutic administration of *BCW* on the RVH hemodynamics was assessed through catheterization (mean right ventricular pressure and mean pulmonary artery pressure (mRVP and mPAP, respectively)). Tissue samples were used to perform histological staining, and confirmatory analyses of mRNA and protein levels were conducted to detect alterations in the mechanisms of RVH in HAHD. The protective mechanism of *BCW* was further verified via cell culture.

**Results:**

*BCW* considerably reduced SuHx-associated RVH, as indicated by macro morphology, HW/BW ratio, FI, mPAP, mRVP, hypertrophy markers, heart function, pathological structure, and myocardial enzymes. Moreover, *BCW* can alleviate the disorder of glucose and fatty acid metabolism through upregulation of carnitine palmitoyltransferase1ɑ, citrate synthase, and acetyl-CoA and downregulation of glucose transport-4, phosphofructokinase, and pyruvate, which resulted in the reduced levels of free fatty acid and lactic acid and increased aerobic oxidation. This process may be mediated via the regulation of sirtuin 3 (SIRT3)-hypoxia-inducible factor 1α (HIF1α)-pyruvate dehydrogenase kinase (PDK)/pyruvate dehydrogenase (PDH) signaling pathway. Subsequently, the inhibition of SIRT3 expression by 3-TYP (a selective inhibitor of SIRT3) can reverse substantially the anti-RVH effect of *BCW* in HAHD, as indicated by hypertrophy marker and serum myocardial enzyme levels.

**Conclusions:**

*BCW* prevented SuHx-induced RVH in HAHD via the SIRT3-HIF1ɑ-PDK/PDH signaling pathway to alleviate the disturbance in fatty acid and glucose metabolism. Therefore, *BCW* can be used as an alternative drug for the treatment of RVH in HAHD.

**Supplementary Information:**

The online version contains supplementary material available at 10.1186/s12906-024-04490-6.

## Introduction

An increasing number of people are being exposed to high altitudes, with more than 81.6 million residing permanently at an elevation > 2500m above sea level; in addition, approximately 40 million individuals are exposed to high altitudes for hours or days [1, 2]. The principal effect of high altitude on humans, which is due to the low atmospheric pressure and subsequent proportional decrease in partial oxygen pressure in the inspired air, involves a reduction in the bioavailability of oxygen in organs, tissues, and cells [3]. Hypobaric hypoxia triggers several mechanisms that compensate for the decrease in oxygen bioavailability. These mechanisms include pulmonary artery vasoconstriction and subsequent pulmonary arterial remodeling [4]; these changes can lead to high-altitude pulmonary hypertension (HAPH) and the development of right ventricular hypertrophy (RVH), right heart failure (RHF), and ultimately death [5]. This disease, which is characterized by pulmonary hypertension and RVH, is called high-altitude heart disease (HAHD).

Despite the vast information that explains RVH via HAPH-induced pressure overload, the contribution of the activation of environmental and neurohumoral factors to the hypertrophic effect and dilation process of RV is worthy of recognition [6]. Chronic and sustained hypoxia can lead to high-altitude polycythemia, increased blood viscosity, and further exacerbation of RVH in HAHD. In addition, myocardial cells are affected by long-term hypoxemia, activation of local renin–angiotensin–aldosterone system, and increased release of catecholamines, which eventually lead to compensatory hypertrophy of the right ventricle (RV) [5, 7]. RVH manifests as an increase in cardiomyocyte size, reappearance of the fetal gene brain natriuretic peptide (BNP), and thickening of the right ventricular wall.

Studies on hypoxic and hypobaric conditions have determined the contributions of oxidative stress, kinase activation, and inflammatory processes to RVH development and its transition to RHF [5, 8]. In addition, triggers of RV dysfunction also include alterations in cellular metabolism, including aerobic glycolysis, fatty acid oxidation, and glucose oxidation [9, 10]. However, knowledge on the metabolic changes and adaptations of RVH in HAHD is limited. Correspondingly, whether RV-targeted metabolic therapies may provide the required additional protective effects necessitates in-depth investigation.

*Bawei Chenxiang Wan (BCW)* is a traditional Tibetan folk medicine formula that includes the following herbs: *Aquilariae Lignum Resinatum*, *Myristicae Semen*, *Choerospondiatis Fructus*, *traverta*, *Olibanum*, *Aucklandiae Radix*, *Chebulae Fructus*, and *Kapok Gossampini Flos* [11]. In clinical practice, it is oral administration after grinding, 1–1.5 g at a time, 2–3 times a day. BCW exhibited activities in anti-acute myocardial ischemia, myocardial ischemia–reperfusion injury, focal cerebral ischemia, and renal ischemia and improved spatial learning and memory in hypoxic rats [12, 13]. As early as 1997, Ma Naowu realized the preventive and curative effects of BCW on high-altitude chronic pulmonary heart disease [14]. With the in-depth scientific research on plateau medicine, Zhuoma Dongzhi and others further elucidated the important role of BCW in the treatment of high-altitudes diseases [15]. Our research group also previously discovered that BCW can improve isoproterenol-induced cardiac hypertrophy by regulating energy metabolism [16]. Therefore whether BCW has an anti-RVH effect on HAHD is worthy of investigation.

In the present study, *BCW* was used in a preclinical rat model of severe RVH in HAHD administered with a bolus of Sugen5416 (Su5416) and subjected to hypoxia exposure (SuHx; 5000 m altitude) for 4 weeks to identify protection mechanisms and possible signaling pathways.

## Materials and methods

### *Bawei chenxiang wan*

*BCW* (CAS18017A) used in our experiment was provided by Tibet Ganlu Tibetan Medicine Co.,Ltd. (Lhasa, Tibet, P. R. China), and we adopted the clinical approach of *BCW* administration (crushing and dissolving with water) for both in vivo and in vitro experiments. *BCW* was administered to rats via an animal gavage needle.

### Chemicals

SU5416(CAS 204005–46-9) and Cell Counting Kit-8 (CCK-8) (CAS C0005) purchased from TargetMol, Trimetazidine (TMZ) was obtained from Servier (Tianjin). 3-TYP (S8628) was purchased from selleck.cn. β-Sitosterol (CAS 83–46–5), luteolin (CAS 491–70–3), Kaempferol (CAS 520–18–3), Costunolide (CAS 553–21–9), Naringenin (CAS 480–41–1),and Quercetin (CAS 117–39–5)were purchased from Chengdu Ruifensi Biotechnology Co., Ltd. (Sichuan, P. R. China).

### Antibodies

**Table:** 

Target antigen	Vendor or Source	Catalog ^#^	Working concentration
Glut4	Immumoway	YT5523	1:1000
Citrate Synthetase	Abcam	Ab129095	1:1000
CTP1α	Abcam	ab234111	1:1000
SIRT3	Immumoway	YT4304	1:1000
HIF1α	Abcam	Ab179483	1: 500
PDK1	Abcam	ab202468	1:1000
PDK2	Abcam	ab68164	1:1000
PDK4	Abcam	ab214938	1:1000
PDHA1	Abcam	ab110334	1:1000
PDHA1(phosphor S293)	Abcam	ab177461	1:1000
β-actin (Mouse)	Cell SignalingTechnology	#3700	1:1000
α-Tubulin(Rabbit)	proteintech	11,224-1AP	1:1500
GAPDH (Mouse)	BeiJing TDY Biotech CO.,Ltd	TDY042C/F	1:1500
Anti-mouse IgG	Cell Signaling Technology	#7076	1:1500
Anti- rabbit IgG	Cell Signaling Technology	#7074	1:1500

### Chromatographic conditions and instrumentation

Methods were performed as previously described [16]. A validation HPLC method was applied onto a Shimadzu (Kyoto, Japan) LC-20AT system. Extraction method of test substance: Weigh 2.0g of sample and put it into volumetric flask, 25ml of methanol and 25ml of water were added separately, and ultrasonicate (power 250W, frequency 33 kHz) for 30 min; stand and filter detection conditions: acetonitrile: 0.2% phosphoric acid water, 0–8 min 9% acetonitrile; 8–20 min 9–60% acetonitrile; 20–55 min 60%; 55–56 min 60%–9%; 56–66 min 9% acetonitrile; detection wavelength: 225nm; flow rate: 1.00 ml/min; and loading volume: 20μL.

### Cell culture

H9_C_2 cells were obtained from the Shanghai Cell Bank, Chinese Academy of Sciences. The cells were cultured in DMEM (SH30022.01B, Hyclone) supplemented with 10% fetal bovine serum (FBS, 10,270–106, Gibco) in an atmosphere containing 5% CO_2_ and 95% air at 37 °C. The medium was replaced every 24h, and the cells were subcultured or cryopreserved when the confluence reached 80%-90%. Under the conditions of cells to be in good condition cells will be diluted to 1 × 10^5^ cells / mL, 100μl/1mL per well, evenly added into 96-well plates / 6-well plates, and cultured for 24h. After the cells reached logarithmic growth stage, the following experimental procedures were performed and conventional culture for 24h. After that, the cells of each group were detected, and the experiment was repeated three times.

### Screening of CoCl_2_ effective concentration and action time

According to relevant literature [17], different concentrations of CoCl_2_ were set to screen the conditions for modeling and cell viability. The specific methods were as follows: When the cells reached the logarithmic phase of growth, the cells were washed with PBS, trypsinized and resuspended. The cells were diluted to a density of 1 × 10^5^ cells /mL, and 100μL of the cell solution in each well was inoculated in a 96-well plate and cultured in a CO_2_ incubator for 24 h. Different concentrations of CoCl_2_ (400μM, 600μM) solution were added, and the cell survival rate was detected by CCK-8 after 12h and 24h of culture.

### Screening of the effective concentration of 3-TYP

Different concentrations of 3-TYP (40μM, 50μM, 60μM) were added to the cell culture medium, and after 12 h of culture, cell RNA and protein were extracted for SIRT3 detection.

### Safety range screening of BCW

After 600μM CoCl_2_ was added into the cell culture medium, different concentrations of BCW (800mg/L, 400 mg/L, 200 mg/L, 100 mg/L) were added to the cell culture medium for 12 h, and the cell survival rate was detected by CCK-8.

### Group of cells

The cell experimental groups were categorized as follows: control group, CoCl_2_ group, CoCl_2_ + BCW group, CoCl_2_ + 3TYP group, CoCl_2_ + 3TYP + BCW group, and CoCl_2_ + 3TYP + TMZ group.

### Animals

Male specific pathogen-free Sprague Dawley (SD) rats aged 2–3 months were obtained from the Xian Jiaotong University Animal Center (SCXK (shaan) 2018–003, Xian, China). Rats were housed in the Xizang Minzu University Laboratory Animal Center with a 12h–12h light–dark cycle. They were fed regular chow and purified water ad libitum. The animal experiment was conducted following the internationally accepted laboratory animal use and care principles. It is reviewed by the Ethics Committee of Xizang Minzu University (Ethics Approval No. 20200–7). The total of 50 SD male rats with an average weight of 180-220g were randomly divided to the following groups (*n* = 10/group): Control, RVH group, RVH + BCW0.8 g kg^−1^d^−1^ group, RVH + BCW0.4 g kg^−1^ d^−1^ group, RVH + TMZ group.

Vascular endothelial growth factor inhibitor SU5416 was injected subcutaneously at a one-time rate of 20mg/kg + chronic hypoxia (SuHx) simulated oxygen chamber (5000m altitude) for 4 weeks, 23h a day, and the remaining 1h was taken out, fed with water and replaced with bedding material.Animals in the control group were raised in an environment outside the cabin (300m local altitude).

### Pulmonary artery pressure, right ventricular pressure was measured by floating catheter

The polyethylene (PE) catheter with transducer at the end was filled with heparin saline. After the air bubble was drained, the transducer was connected to the Power Lab multi-channel electrophysiological recorder, and the pressure waveform curve was simultaneously recorded by Lab Chart 8.0 software. After the rats were anesthetized by intraperitoneal injection of 2% sodium pentobarbital (60 mg/kg), the rats were fixed on the anatomical plate in the supine position. Preserved skin, separated the right subcutaneous tissue, expose the right external jugular vein and dissociated.The PE tube was inserted through the incision after cutting out the V-shaped incision, and the waveform of venous pressure was observed on the recorder. After that, mRVP and mPAP were measured in each group.

### Measurement of right ventricular hypertrophy index in rats

Following hemodynamic parameter measurements, the weight of the whole heart was obtained. The right ventricle was then separated from the left ventricle plus septum (LV + Septum). RV hypertrophy was assessed by the Fulton index (FI; ratio of RV to LV + septum weights). All collected tissues were either flash frozen in liquid nitrogen or fixed with 4% paraformaldehyde at room temperature for further analysis.

### Pathological HE staining of myocardial tissue

The maximal cross sections of the hearts were perfused with normal saline and then fixed in 4% paraformaldehyde for 24 h. Serial paraffin sections about 0.4μm thick were prepared by rinsing with running water, dehydration, transparency, and embedding in paraffin. Paraffin sections were routinely dehydrated. HE staining was performed with hematoxylin for 10 min at room temperature, followed by a rapid rinse with tap water for 30-60s. The cells were differentiated with 1% alcohol hydrochloride for 1s, and then rinsed with tap water for 60s. The cells were stained with eosin for 5–10 min at room temperature. Gradient ethanol dehydration; Xylene transparent; Seal with neutral gum. Finally, the sealed paraffin sections were observed under a light microscope and photographed.

### ELISA protein analysis

Rat ELISA kits (BNP, CK-MB, LDH, PFK, acetyl-CoA and pyruvate) from mlbio (Shanghai, China) was used accordingly to the manufacturer’s recommendation. Briefly, 50μL of each standard and sample was added into each well of a 96-well plate, followed by 100μL of 1X HRP–streptavidin solution and incubated for 1h at 37℃ while gently shaking. After 1 h of shaking at 37℃, washed 5 times, and 50μL A, B substrate Reagent added. After 15 min of 37℃ incubation in the dark and the addition of 50μL of Stop Solution to each well, the plate was immediately read at 450nm.

### Determination of lactic acid and free fatty acids

The arterial blood samples harvested from all groups were centrifuged at 3,000 rpm for 10 min at 4 °C. The plasma supernatant was obtained and stored at − 80°C. Subsequently, operational testing was performed according to the relevant kit instructions. Free fatty acids (FFA), Lactic acid (LD) obtained from Nanjing jiancheng Bioengineering Institute, Nanjing, China.

### Real-time quantitative polymerase chain reaction

According to the manufacturer’s instructions, total RNA from rats heart tissue was extracted with Magzol reagent (MGBio, Shanghai, China)(RNA extraction methods are described in the Supplementary Material). Reverse transcription was performed using the two-step RT kit (Vazmye, China). PCRs were performed with the SYBR Green Quantitative PCR kit using the following primers: PDHA1, PDK1, PDK2, PDK3, PDK4, SIRT1-7 (sangon Biotch, Shanghai, China), Quantification was performed using the efficiency-corrected − 2^ΔΔCT^ method with the housekeeping gene GAPDH as endogenous control. Data were presented as folding change over the control group.The sequences of target genes and internal reference primers are as follows:

**Table:** 

Target Gene	Sequence
PDHA1-Rat	FORWARD: CTGAGGGTAGATGGAATGGA REVERSE: TGGTAGCGGTAAGTCTGTAG
GAPDH-Rat	FORWARD: AGTTCAACGGCACAGTCAAGGC REVERSE: CGACATACTCAGCACCAGCATCAC
PDK1-Rat	FORWARD: CGAGACGGCTTTGTGATTT REVERSE: GAGATGGGACGGAACATAAAC
PDK2-Rat	FORWARD: GACTTGCAGCTCTTCTCTATG REVERSE: CAGGCAGACTTGTTGTAGAC
PDK3-Rat	FORWARD: CCGCTCATCCGAAACATATAG REVERSE: TTCTGGAGCTACCAGGTAATA
PDK4-Rat	FORWARD: GAACCAGCACATCCTCATATT REVERSE: CTCAAAGGCATCTTCGACTAC
SIRT3-Rat	FORWARD: CTGCGGCTCTACACACAGAA REVERSE: CATCACGTCAGCCCGTATGT
β-actin-Rat	FORWARD: TCGTGCGTGACATTAAAGAG REVERSE: ATTGCCGATAGTGATGACCT

### Western blotting

Western blotting assay was performed as previously described [18]. Protein extraction methods are described in the Supplementary Material. Equal amount of proteins (30mg of total proteins) from rats heart tissue was separated by 10% sodium dodecyl sulfate–polyacrylamide gel electrophoresis (SDS-PAGE), and then transferred onto polyvinylidene fluoride (PVDF) membranes (Millipore). The membranes were incubated with primary antibodies overnight at 4 °C, followed by incubating with appropriate horseradish peroxidase–conjugated secondary antibodies at room temperature for1h. The blots were developed with super signal chemiluminescent substrate (Diyibio, Shanghai, China) and detected by the ChemiDocTM XRS + (Bio-Rad, CA, United States). The intensities of the blots were quantified by Quantity One software and α-Tubulin,β-actin or GADPH served as a loading control.

### Data analysis

The statistical analysis was conducted using GraphPad Prism 8 (GraphPad Software). The data were presented as mean ± SE. Statistical analysis among various groups was performed by one-way analysis of variance (ANOVA) with the Bonferroni post hoc test. In all cases, difference between groups was statistically significant when *p* < 0.05.

## Results

### Quality control analysis of BCW

*BCW* methanol and water extracts were analyzed by high-performance liquid chromatography, and the main components were quantified. A standard curve of 225 nm was used to quantify the chromatograms of six Chinese herbs (Figure S1**)**, and the six main components of *BCW* were detected and calculated separately using the formula (content (mg/g) = peak area of sample/peak area of reference substance multiplied by concentration of reference substance/concentration of sample). The six main components of *BCW* comprised β-sitosterol (38.90 mg/g), luteolin (0.13 mg/g), kaempferol (0.09 mg/g), costunolid (0.24 mg/g), naringenin (0.04 mg/g), and quercetin (7.23 mg/g) (Table S1).

### *BCW* intervention ameliorates pulmonary arterial pressure and right ventricular dysfunction induced by SuHx

**Fig. 1:**
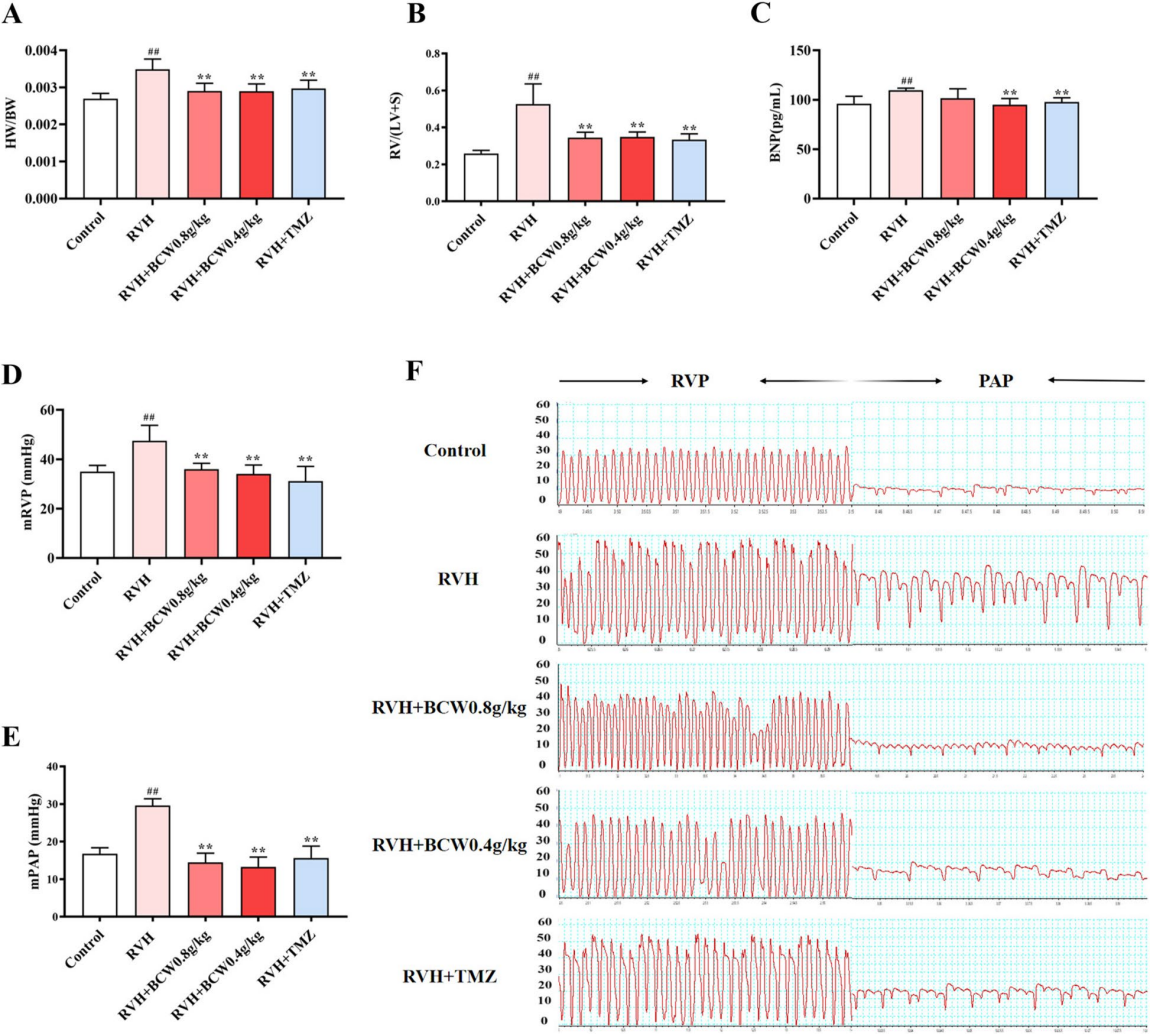
*BCW* intervention ameliorates pulmonary arterial and right ventricular dysfunction mediated by SuHx. **A** and **B** Heart weight/body weight ratio and Fulton index. **C** BNP expression was analyzed by ELISA kit. **D**, **E** Mean right ventricular pressure and mean pulmonary artery pressure. **F** Pressure waveforms of the right ventricle and pulmonary artery from the right ventricular cannulation. Results are expressed as mean ±SE: ^#^*p*<0.05, ^##^*p*<0.01 vs control; ^*^p<0.05vs RVH, ^**^p<0.01 vs RVH, *n* = 10 in each group

Rats were dosed with Su5416 and subjected to SuHx for 4 weeks to establish a model of RVH in HAHD. Hypertrophic indexes and pressure were measured in SuHx-induced rats to confirm establishment of the model, which was indicated by the increased heart weight-to-body-weight ratio (HW/BW) and Fulton's index (FI) (RV/(left ventricle + septum weight) in the critical assessment of RVH (Fig. 1A–B). In addition, right heart catheterization is the gold standard for the diagnosis of pulmonary hypertension. As appreciated from the pressure waveform diagram (Fig. 1F)**,** SuHx not only increased RV pressure (Fig. 1D) but was accompanied with an increase in mPAP (Fig. 1E). Administration of *BCW* markedly reduced the SuHx-induced increases in RV weight (HW/BW), FI and pressure (mRVP), and attenuation in mPAP. Further, a remarkable improvement in the SuHx-induced increase in the cardiac hypertrophy marker BNP was observed, which supports the restoration of RV function (Fig. 1C). These results demonstrate that *BCW* can significantly improve RVH.

### *BCW* attenuated SuHx-induced myocardial injury in the RVH

**Fig. 2:**
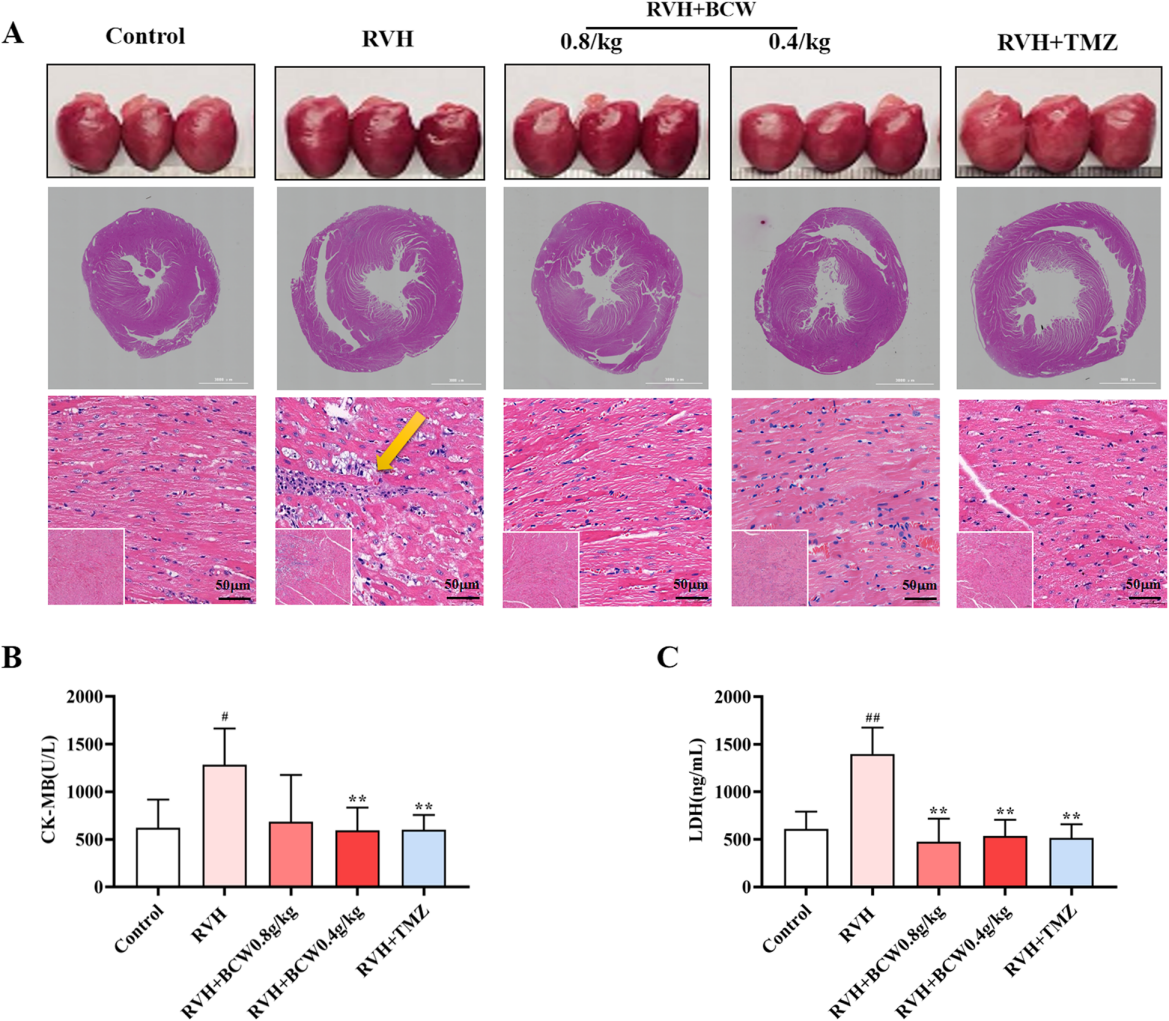
*BCW* attenuated SuHx-induced myocardial injury in the RVH. **A** Gross morphology of the heart and cross-sectional views of hematoxylin and eosin, A = 5000 μM,100 μM and 50 μM (bottom) scale ruler, magnified (5000 × , 100 × and 400 ×). **B** and **C** Serum CK-MB and LDH levels were measured using ELISA kits, respectively. Results are expressed as mean ± SE: ^#^*p* < 0.05, ^##^*p* < 0.01 vs control; ^*^p < 0.05vs RVH, ^**^p < 0.01 vs RVH, *n* = 10 in each group

The morphology of the myocardium was further observed via hematoxylin–eosin staining of the premise of the general morphology of the heart. The RVH group revealed thickening of the ventricular wall, enlargement of cardiomyocyte, degeneration and necrosis of myocardial fibers, and proliferation of fibrous tissue in the cross section of the heart (Fig. 2A). Notably, *BCW* alleviated these SuHx-induced changes in myocardial morphology. In addition, serum myocardial enzyme levels were measured using commercial kits. As shown in Fig. 2B-C, lactate dehydrogenase (LDH) and creatine kinase-MB (CK-MB) levels were increased considerably in the RVH group but were substantially inhibited by *BCW*. Combined with our data, these results suggest that *BCW* intervention may have contributed to the enhanced RV function recovery through attenuation of RVH. However, this process can be improved by *BCW*.

### Association between *BCW* and fatty acids/glucose metabolites in RVH

**Fig. 3:**
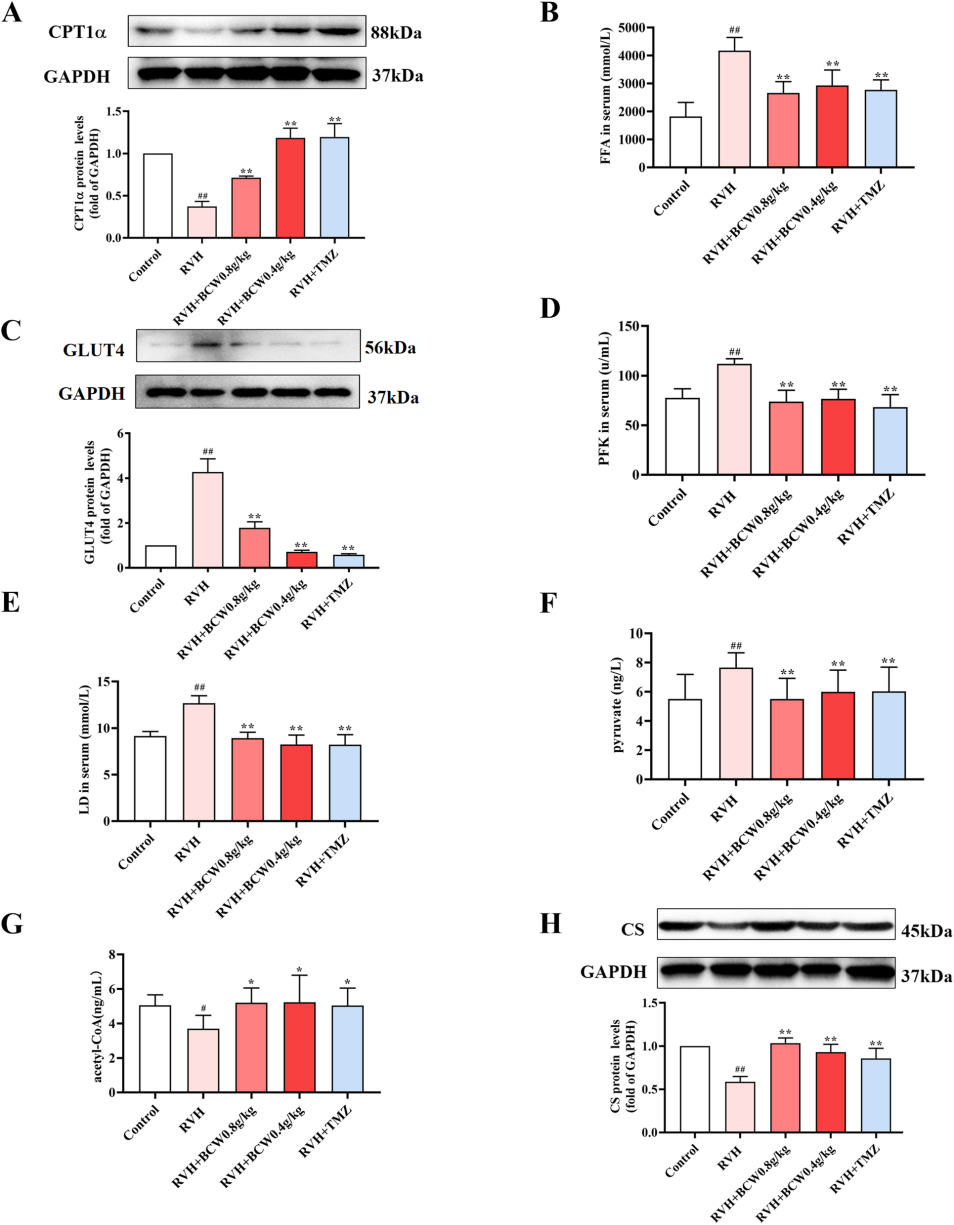
Association between *BCW* and fatty acid/glucose metabolites in RVH. **A** The protein expression of CPT-1α was determined by immunoblotting. Densitometric analyses of (**A**) immunoblots are presented as ratios of controls. **B**, **D**-**G** kits were used to measure serum FFA, PFK, LD, pyruvate, and acetyl-CoA levels (**C** and **H**) The protein expression of GLUT4 and CS were determined by immunoblotting. Densitometric analyses of (C and H) immunoblots are presented as ratios of controls. Results are expressed as mean ± SE: ^#^*p* < 0.05, ^##^*p* < 0.01 vs control; ^*^p < 0.05 vs RVH, ^**^p < 0.01 vs RVH, *n* = 10 in each group

To gain insights into the protective mechanism of *BCW* in RVH, we focused on the free fatty acid (FFA) and carnitine palmitoyltransferase1ɑ (CPT1α) of the heart to illustrate the fatty acid metabolism underlying the anti-RVH effect of *BCW* on HAHD. Our experimental results show the increased FFA levels in the RVH group, which was notably inhibited by *BCW* (Fig. 3B). This finding, which indicates reduction in fatty acid utilization in the myocardium, may be related to the decreased protein level of CPT1ɑ and reduced fatty acid entry into mitochondria for oxidation (Fig. 3A). Metabolic changes occurred in RVH, in which fatty acid metabolism rates decreased, but such alterations can be elevated by *BCW*.

Furthermore, glucose transport-4 (GLUT4), a glucose transporter responsible for glucose uptake, was detected by Western blotting. As presented in Fig. 3C, the protein levels of GLUT-4 was decreased by SuHx, and *BCW* considerably reversed these changes. Moreover, a key kinase enzyme phosphofructokinase (PFK) that phosphorylates fructose 6-phosphate in glycolysis was enhanced treated by SuHx, which were decreased by *BCW*, especially at the dose of 0.8 and 0.4 g kg^−1^ day^−1^ groups (Fig. 3D). Similar observations were made regarding pyruvate (Fig. 3F). These results, together with the increased FFA and decreased CPT1α, imply that RVH in HAHD can cause a relative increase in glucose uptake compared with that of fatty acid. However, whether this condition denotes an increase in glycolysis or glucose oxidation requires further exploration.

In addition, lactic acid (LD), a glycolysis product, was forcefully elevated in serum and notably inhibited by *BCW* (Fig. 3E). Meanwhile, acetyl-CoA, as the primary step in aerobic glucose metabolism, was reduced in the RVH group, along with citrate synthase (CS), which is responsible for catalyzing the first reaction of the citric acid cycle (Krebs cycle or tricarboxylic acid (TCA) cycle) (Fig. 3G–H). Thus, the substantial reduction of CS and acetyl-CoA and augmentation of LD in the RVH group prove the suppression of TCA and increase in glycolysis, which was reversed by *BCW* (Fig. 3G–H). Collectively, the improvement of these data are supportive of the positive effect of *BCW* on RVH in HAHD, which led to the enhanced RV function and its possible dependency and unison with fatty acid/glucose metabolite protection.

### SuHx have increased pyruvate dehydrogenase kinase (PDK) expression and decreased pyruvate dehydrogenase (PDH) activity which is reversed by *BCW*

**Fig. 4:**
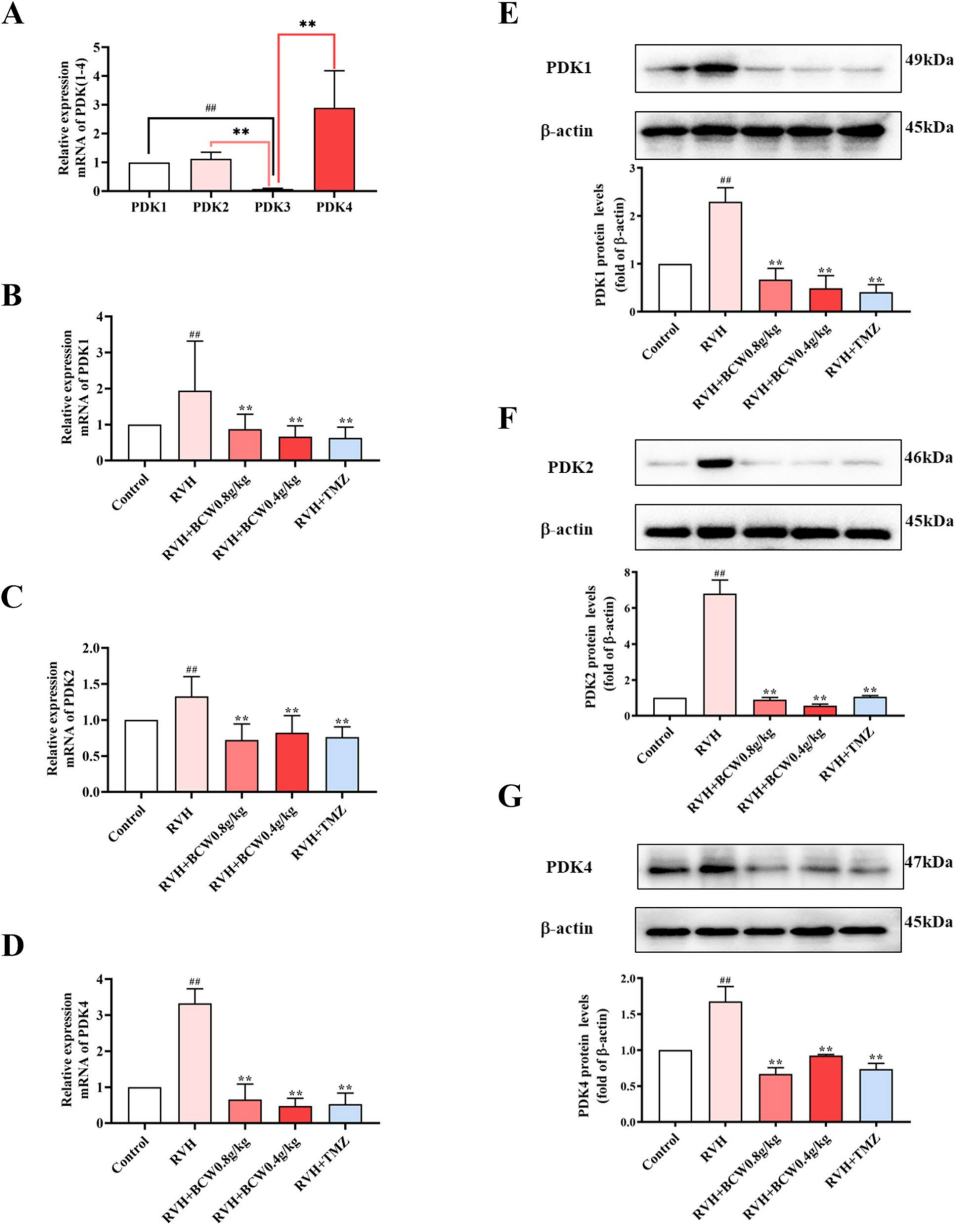
Effect of *BCW* on mRNA and protein expression levels of PDK. **A**-**D** Real-time quantitative PCR analysis of PDKI-4 mRNA expression in normal rat cardiomyocytes. The mRNA expression was normalized by GADPH. **E**–**G** The protein expressions of PDK1, PDK2 and PDK4 in vivo were detected by western blotting. **E**–**G** Density analysis of western blots was expressed as the ratio of controls. Results are expressed as mean ± SE: ^#^*p* < 0.05, ^##^*p* < 0.01 vs control; ^*^p < 0.05 vs RVH, ^**^p < 0.01 vs RVH, *n* = 10 in each group

PDK1-4, a serine-specific kinase with a range of 45–48k Da, plays a major role in phosphorylation and inactivation of PDH-E1ɑ. We first examined the expressions of PDK1, PDK2, PDK3, and PDK4 in normal rat cardiomyocytes through real-time polymerase chain reaction. As shown in Fig. 4A, PDK1, PDK2, and PDK4 are highly abundant in cardiomyocytes. As shown in Fig. 4B–G, the mRNA and protein levels of PDK1, PDK2, and PDK4 were considerably increased in the RVH group, but these changes are *BCW* significantly reversed.

**Fig. 5:**
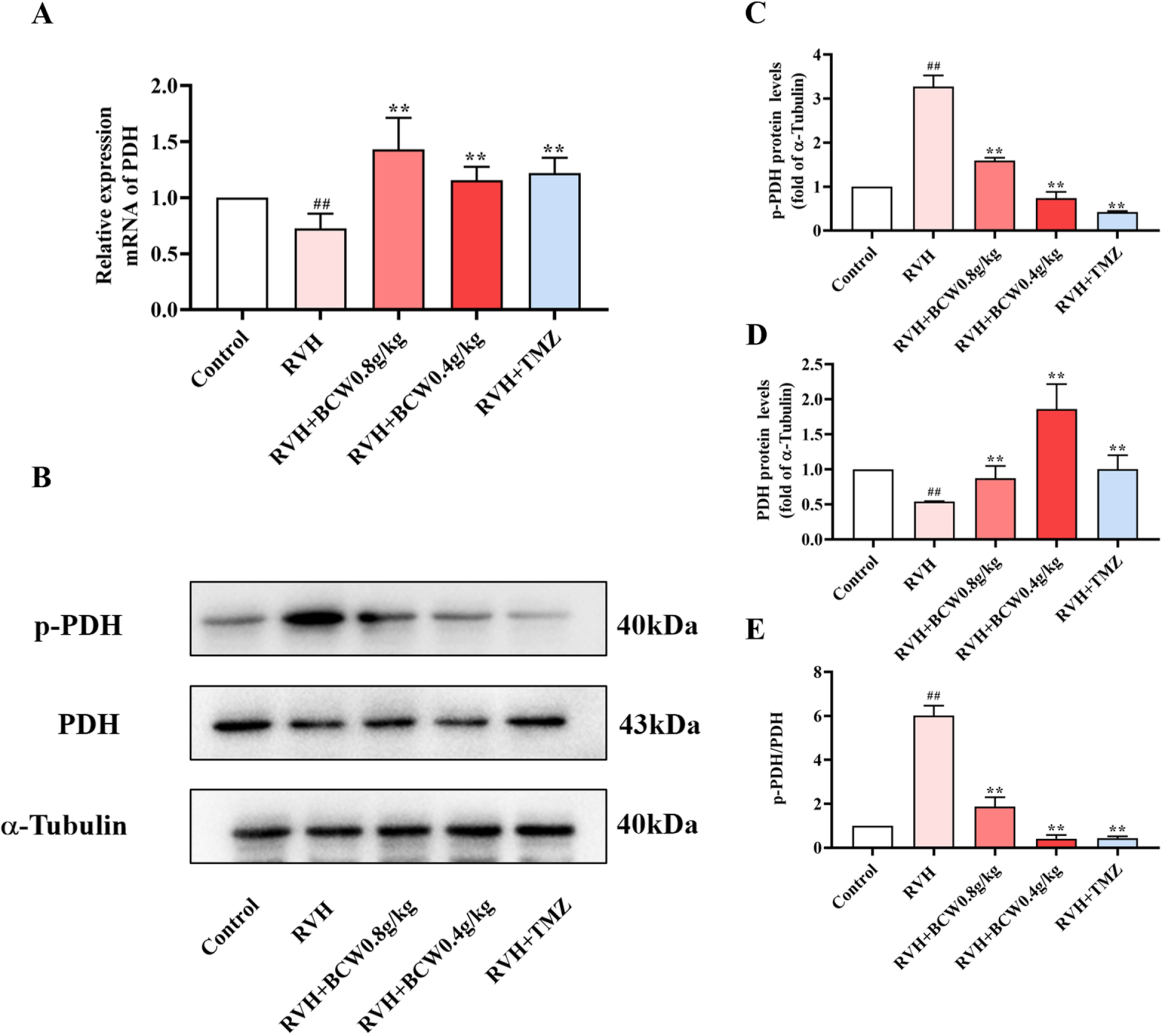
Effect of *BCW* on mRNA and protein expression levels of PDH. **A** Real-time quantitative polymerase chain reaction was used to analyze the mRNA level expression of PDH. The mRNA expression was normalized by GADPH. **B** The protein expression of p-PDH and PDH in vivo was detected by western blotting. **C**, **D**, **E** Density analysis of western blotting was expressed as the ratio of control. Results are expressed as mean ± SE: ^#^*p* < 0.05, ^##^*p* < 0.01 vs control; ^*^p < 0.05vs RVH, ^**^p < 0.01 vs RVH, *n* = 10 in each group

PDH catalyzes the oxidative decarboxylation of pyruvate to form acetyl-CoA and carbon dioxide, links glycolysis and TCA cycle and acts as a “gatekeeper” in glucose oxidation to maintain glucose homeostasis. Then, we discovered that phosphorylated PDHE1-α subunit (p-PDH) and p-PDH/PDH expression ratio were increased, which was effectively reversed by *BCW* (Fig. 5). Notably, PDK-mediated inactivation of PDH phosphorylation, which can be ameliorated by *BCW*, can inhibit the TCA cycle and fatty acid metabolism and increase glycolysis in cardiomyocytes. This result was associated with increased FFA, LD, PFK, GLUT4, and pyruvate levels and decreased levels of CPT1ɑ, acetyl-CoA, and CS.

### Expressions of sirtuin 3 (SIRT3) and HIF1α in the heart of SuHx-induced RVH rats

**Fig. 6:**
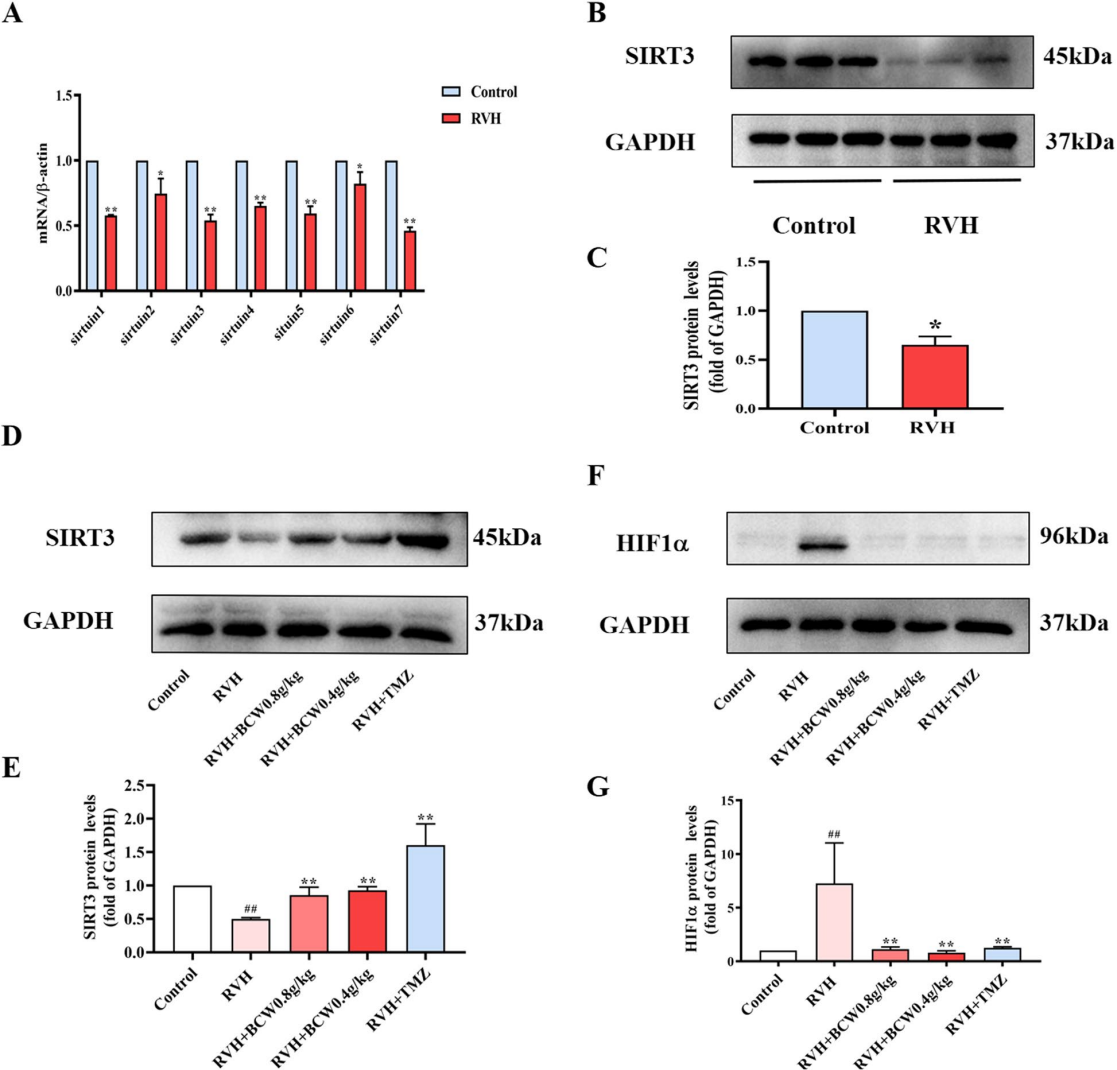
Effect of *BCW* on mRNA and protein expression levels of SIRT3 and HIF1α. **A** Real-time quantitative polymerase chain reaction analysis of SIRT1-7 mRNA levels in vivo. GADPH was used to normalize mRNA expression. **B** The protein expression of SIRT3 in vivo was detected by Western blot. Densitometric analysis of **C** immunoblots is presented as the ratio of the control. **D** and **F** protein expression of SIRT3 and HIF1α in vivo was detected by Western blotting. Densitometric analysis of (**E** and **G**) immunoblots is presented as the ratio of the control. Results are expressed as mean ± SE: ^#^*p* < 0.05, ^##^*p* < 0.01 vs control; ^*^p < 0.05vs RVH, ^**^p < 0.01 vs RVH, *n* = 10 in each group

The SIRT protein family plays various roles in metabolism, and it is considered a potential therapeutic target for cardiovascular disease. Studies have shown that SIRT3 is highly expressed in metabolically active organs, such as the heart, it can modulate proteins involved in metabolic function regulation, including fatty acid metabolism and glucose metabolism, which is our study's focus. As presented in Fig. 6A–C, SIRT3 mRNA and protein levels were markedly reduced in the RVH group. On the contrary, the protein expression of SIRT3 was reversed after *BCW* intervention **(**Fig. 6D–E**)**.

Hypoxia-inducible factor (HIF1α) is a major transcription factor for genes related to oxygen homeostasis, and it has been described as a key regulator of hypoxia-induced diseases. To investigate whether HIF1α is activated in RVH in HAHD, we determined its expressions through Western blotting. As shown in Fig. 6F–G, HIF1α level in cardiomyocytes was considerably decreased after *BCW* treatments (0.8 and 0.4 g kg^−1^ day^−1^). These observations suggest the activation of SIRT3and suppressed HIF1α by the response of RVH in *BCW* intervention.

Thus, SIRT3 can regulate the biological function of HIF1α via targeted acetylation, which mediates metabolic reprogramming. Notably, future studies on the specific regulatory mechanism need to be conducted.

### *BCW* inhibits cellular hypoxia via the SIRT3-HIF1α–PDK/PDH pathway in H9c2 cell hypoxia model induced by CoCl_2_

**Fig. 7:**
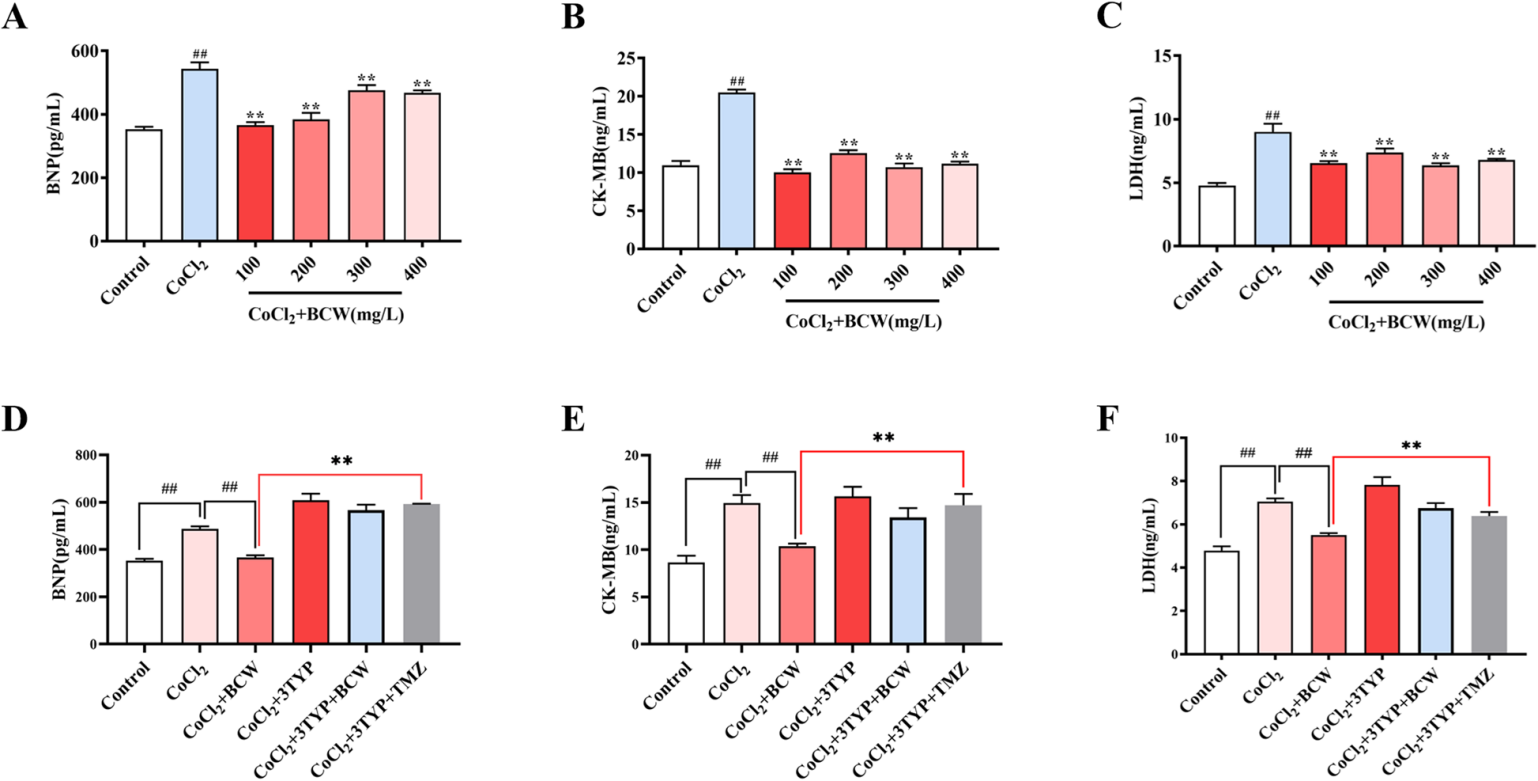
Protective effect of *BCW* on hypoxic induced by CoCl_2_. **A**-**C** The protective effect of *BCW* on CoCl_2_-induced hypoxic cell model was detected by ELISA kits (BNP, CK-MB, LDH). Results are expressed as mean ± SE: ^#^*p* < 0.05, ^##^*p* < 0.01 vs Control; ^*^p < 0.05, ^**^p < 0.01 vs CoCl_2,_ *n* = 3. **G**, **H**, **I** ELISA kits (BNP, CK-MB, LDH) were used to detect the protective mechanism of *BCW*. Results are expressed as mean ± SE: ^#^*p* < 0.05, ^##^*p* < 0.01 vs Control; ^#^*p* < 0.05, ^##^*p* < 0.01 vs CoCl_2_; ^*^p < 0.05, ^**^p < 0.01 vs BCW + CoCl_2_, *n* = 3

To further verify our experimental results on the mechanisms of SIRT3 and HIF1ɑ regulation, we used H9c2 cells to establish an in vitro hypoxic model to simulate RVH in HAHD. Based on half maximal inhibitory concentration, 600 µmol for 12h is the best concentration and time for CoCl_2_ modeling (Figure S3D), and 100 mg/L is the safest range and most protective concentration for *BCW* throng the cell viability, BNP, CK-MB, and LDH (Figures S3E-S3F and Fig. 7A-C). These observations suggest that *BCW* has a protective effect against CoCl_2_-induced hypoxia in vitro.

To confirm that the elevated levels of SIRT3 were regulated by *BCW*, we inhibited SIRT3 with 3-TYP, a selective inhibitor of SIRT3. The mRNA and protein expression levels demonstrated 50 µmol for 12 h as the optimal inhibitory concentration and time for 3-TYP (Figures S3A–S3C**)**. Moreover, 3-TYP reversed the protective effect of *BCW* (Fig. 7D–F), as indicated by the results on BNP, CK-MB, and LDH.

**Fig. 8:**
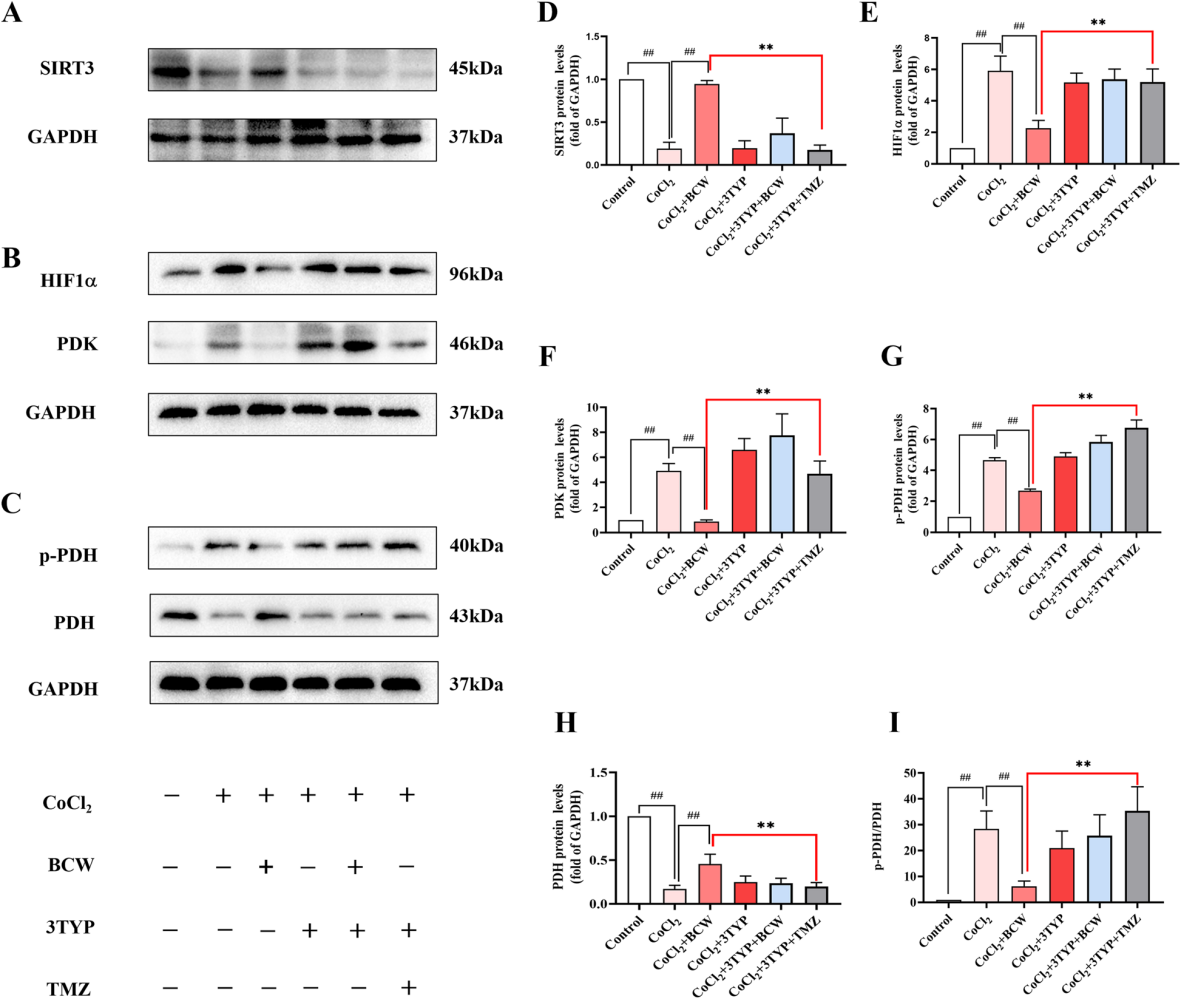
*BCW* inhibits CoCl_2_-induced injury in H9c2 cells through the SIRT3-HIF-1α -PDK/PDH pathway. **A**, **B**, **C** In vitro protein expression of SIRT3, HIF-1α, PDK, p-PDH, and PDH was determined by immunoblotting. Densitometric analyses of (**D**, **E**, **F**, **G**, **H**, **I**) immunoblots are presented as ratios of controls. Results are expressed as mean ± SE: ^#^*p* < 0.05, ^##^*p* < 0.01 vs Control; ^#^*p* < 0.05, ^##^*p* < 0.01 vs CoCl_2_; ^*^p < 0.05, ^**^p < 0.01 vs BCW + CoCl_2_, *n* = 3

The results are similar to our in vitro observations. Western blotting was applied to CoCl_2_-induced hypoxia in H9c2 cells to validate the correlation between SIRT3 and the HIF1α/PDK/PDH pathway in vitro. Compared with that of the control group, the expression of SIRT3 was considerably decreased, and those of HIF1α, PDK, p‐PDH, and p-PDH/PDH were increased substantially in the CoCl_2_ and CoCl_2_ + 3-TYP groups **(**Fig. 8**)**. Meanwhile, *BCW* can activate SIRT3 and downregulate the expression levels of HIF1α, p‐PDH, and p-PDH/PDH. However, the inhibition of SIRT3 expression notably reversed the effect of *BCW*. These data suggest that *BCW* may attenuate RVH via the partial inhibition of HIF1α/PDK/PDH.

## Discussion

HAHD is characterized by pulmonary hypertension accompanied with RVH or RVF. Despite the new developments in HAHD therapy, cure for RVH is currently lacking, and novel therapies are desperately needed. This study revealed the protection conferred by *BCW* to SuHx- and CoCl_2_-induced H9c2 hypoxia cells in vivo and in vitro, as shown by the improved heart function, macro and micromorphology, and cardiac hypertrophy markers, which are associated with the improved fatty acid and glucose metabolism attained by activation of the SIRT3-HIF1α-PDK/PDH signaling pathway. All these pieces of evidence show that *BCW* may be a potential option for the protection of RVH in HAHD.

More importantly, the TMZ compound plays a crucial role in regulating energy metabolism and is extensively utilized in the management of angina pectoris, ischemic heart disease, and heart failure. It has been discovered by Fillmore that modulating cardiac energy metabolism through reducing fatty acid oxidation or enhancing glucose oxidation can significantly improve the functioning of both ischemic and failing hearts [19]. Therefore, TMZ was considered as a more appropriate positive control for this study [20–22].

*BCW* powder has a protective effect on myocardial ischemia [12, 13]. In addition, a previous survey showed that *BCW* ameliorates cardiac hypertrophy via the activation of AMP-activated protein kinase/peroxisome proliferator-activated receptor-α signaling pathway, which improves energy metabolism [16]. Hence, we discuss the effect and mechanism of *BCW* for RVH in HAHD. Our study revealed that *BCW* treatment not only decreased the size of the heart and levels of hypertrophy markers but also alleviated histopathological changes and impairment of cardiomyocytes (Figs. 1A–C and 2). Furthermore, *BCW* improved cardiac function by decreasing the ventricular wall thickness, RV pressure, and mPAP (Fig. 1D–F), consistent with the results of previous studies [23]. Altogether, we demonstrated that *BCW* can protect SuHx-induced RVH in HAHD, but its specific mechanisms are still unclear.

In our study, long-term metabolic disorder with glycolysis as the main energy supplying mechanism can induce myocardial cells to undergo chronic remodeling in terms of structure and function, together with the decrease in fatty acid metabolism in cardiomyocytes, which eventually leads to RVH in HAHD. In addition, *BCW* treatment can relieve glucose and fatty acid metabolism disorder through upregulation of CPT-1α, CS, and acetyl-CoA and downregulation of GLUT4, PFK, and pyruvate, which result in decreased FFA and LD levels and increased aerobic oxidation. Thus, *BCW* can play a role in cardioprotection.

This metabolism disorder mainly involves the inactivation of PDH phosphorylation. PDK-mediated inactivation of PDH can inhibit the TCA cycle and increase glucose uptake in cardiomyocytes, which results in a compensatory increase in glycolysis and increased lactate production. This pattern of enhanced glycolysis and impaired glucose oxidation has been previously observed in patients with pulmonary hypertension and RVH and animal models [9, 24]. Such finding is consistent with our conclusion that disorders of glucose and fatty acid metabolism occur with RVH in HAHD. In addition, PDK,which is an important HIF1α-targeted gene can be regulated by SIRT3 [25, 26]. Such finding is consistent with our conclusion that disorders of glucose and fatty acid metabolism occur with RVH in HAHD.SIRT3 is highly expressed in the myocardium [17, 27–29], and can regulate fatty acid oxidation and the TCA cycle to improve energy metabolism disorders [30]. *SIRT3* knockout mice showed reduced myocardial energy production, which further impaired cardiac function and metabolism and led to cardiac remodeling and reduced function [31]. Similar findings have been discovered in our study, a low expression in SuHx-induced rat model and CoCl_2_-induced H9c2 cell hypoxia model (Figs. 6A-C and S2).On the contrary, the expression of SIRT3 can reduce the apoptosis of cardiomyocytes in a cardiac hypertrophy model [27]. This finding is similar to the results of our study (Fig. 6). SIRT3 can also mediate metabolism in the SuHx-induced rat model by altering the biological function of HIF1α via targeted acetylation regulation [29, 31].

In summary, consistent with our experimental results showing the increased SIRT3 expression and reduced HIF1α expression in SuHx-induced RVH in HAHD (Fig. 6D–G). Thus, SIRT3 may be an upstream regulator of HIF1α expression.PDK can be induced by HIF1α, and it deactivates the PDH complex through the phosphorylation of the PDHɑ1 subunit at S293. As result, the conversion of pyruvate to acetyl-CoA is inhibited, which reduces the TCA cycle, oxidative phosphorylation, and ATP production. This condition is consistent with the decreased acetyl-CoA and PDH and increased PDK, p-PDH, and p-PDH/PDH levels in the RVH group in our study (Figs. 4, 5, and 6). Therefore, targeting of the SIRT3 gene and regulation of downstream metabolism signaling pathways may be a novel strategy for the treatment of RVH in HAHD.

The use of *BCW* in the treatment of RVH in HAHD may be a novel strategy. In SuHx-induced RVH model and CoCl_2_-induced H9c2 cell hypoxia model, *BCW* treatment can upregulate SIRT3 and PDH, downregulate PDK, p-PDH, and p-PDH/PDH, and inhibit myocardial injury markers (BNP, CK-MB, and LDH) (Fig. 7D-F). Although SIRT3 was inhibited by 3-TYP inhibitors, the antihypertrophic effect of *BCW* decreased (Fig. 8). Therefore, *BCW* prevents RVH in HAHD via the SIRT3-HIF1α-PDK/PDH pathway to partly alleviate the disturbance in glucose and fatty acid metabolism.

Further, future RV-targeted therapies may focus on the role of SIRT3 and its potential relevance in the RVH associated with HAHD. Our findings shed further light on the cardiopulmonary effects of *BCW* and open up opportunities for future investigations on myocardium-specific protective pathways activated by *BCW* and its ingredients, future expansion of RV-targeted therapeutic modalities, and the the possible multiuse of *BCW*.

This paper focused on the systematic investigation of the protective mechanism of *BCW* against RVH in HAHD. However, the complex composition of the drug caused difficulty in determining whether its effect is attributed to the overall formulation or a specific component. Therefore, further research in this area is warranted.

## Conclusion

Our results demonstrated that *BCW* can repress SuHx-induced RVH in HAHD through improvement of glucose and fatty acid metabolism disturbance. In addition, *BCW* protected against RVH in HAHD via the activation of the SIRT3-HIF1α-PDK/PDH pathway and amelioration of myocardial glucose and fatty acid metabolism. These findings provide notable support for the protection provided by *BCW* during RVH in HAHD.

### Supplementary Information

Supplementary Material 1.

Supplementary Material 2.

Supplementary Material 3.

## Data Availability

A part of datasets are included in this published article and its supplementary files. The algorithms used to process the data are available from the corresponding author on reasonable request.

## Abbreviations

RVH: Right ventricular hypertrophy
HAHD: High altitude heart disease
BCW: Bawei Chenxiang Wan
HAPH: High-altitude pulmonary hypertension
RV: Right ventricle
SuHx: Sugen5416-chronic hypoxia
SU5416: Sugen5416
mPAP: Mean pulmonary arterial ressure
mRVP: Mean right ventricular pressure
PDK: Pyruvate dehydrogenase kinase
PDH: Pyruvate dehydrogenase
p-PDH: Phosphorylated PDH
HW/BW: Heart weight to body weight ratio
RV/(LV + S): Ratio of RV to LV + septum weights
FFA: Free fatty acids
LD: Lactic acid
LDH: Lactate Dehydrogenase
CK-MB: Creatine Kinase-MB
CPT1α: Carnitine palmitoyltransferase1ɑ
FFA: Free fatty acids
PFK: Phosphofructokinase
CS: Citrate synthase
HIF1α: Hypoxia-inducible factor 1α
