# Supplementary material for: Bawei Chenxiang Wan ameliorates right ventricular hypertrophy in rats with high altitude heart disease by SIRT3-HIF1α-PDK/PDH signaling pathway improving fatty acid and glucose metabolism
Source: BMC Complement Med Ther. 2024 May 15;24:190. doi: 10.1186/s12906-024-04490-6 (PMC11094862; doi:10.1186/s12906-024-04490-6)
Supplement: Supplementary file 1 — Supplementary Material 1. [file 12906_2024_4490_MOESM1_ESM.pdf]

Supplementary Material (The method of RNA and protein extraction)

The method of RNA extraction

1. Cells: Discard the medium in the petri dish, wash it with pre-cooled PBS for 3 times, add 1ml of pre-cooled RNAiso Plus, gently blow the cells to make them fall off, and transfer the liquid to a new 1.5ml EP tube.

2. Tissue: 20mg myocardial tissue was taken, 1ml RNAiso Plus was added, homogenized on ice, 12000g at 4℃, centrifuged for 10min, and supernatant was transferred to a new 1.5ml EP tube.

3. Add 1/5 volume (200μl) of chloroform into the above cell (tissue) solution, violently shake for 15s until the solution is fully emulsified, and stand at room temperature for 5min, stratification occurs.

4. Centrifuge at 4℃ at 12000g for 15min. Carefully transfer the supernatant to a new 1.5ml EP tube, add an equal volume of isopropyl alcohol precipitated RNA, gently reverse the EP tube and fully mix, and leave for 10min at room temperature.

5. Centrifuge at 4℃ at 12000g for 10min. After abandoning the supernatant, a small amount of white precipitate was observed at the bottom of the EP tube.

6. Slowly add 75% ethanol prepared with 1ml-20 ℃ pre-cooled DEPC treated water into the EP tube, gently upside down, clean and precipitate.

7. Centrifuge at 12000g at 4℃ for 5min, discard the supernatant, open the centrifugal tube cover, and dry in the fume hood for about 10min until the white precipitation

disappears.

8. Take about 15μL of DEPC treated water to dissolve and precipitate. The RNA was fully dissolved in 58℃ water bath for 10min. Flick the EP tube, instantaneously dissociate, and store the mixed RNA solution in the refrigerator at -80℃ or conduct subsequent experiments.

9. The microquantifier Nanodrop 2000 was used for quantification and RNA quality check.A value of A260/280 between 1.8-2.0 indicates that the RNA purity is good, less than 1.8 indicates that the extracted RNA is mixed with impurities such as protein, and more than 2.0 indicates that the RNA is degraded.

The method of protein extraction

1. Tissue: Weigh myocardial tissue 15-20mg on ice, add 300μL RIPA lysate (including protease inhibitor cocktail), and add 1mM PMSF before clinical use. After the tissue was cut into small pieces of $1mm^3$ with surgical scissors on the ice, the homogenizer was used to mechanically homogenize the tissue for 1min, and the homogenizer stopped for 10s after 20 seconds to prevent heat accumulation.

2. Cells: The medium was discarded, the cells were washed twice with pre-cooled PBS buffer, 40μL cell lysate was added, the cells were gently scraped off and transferred to another new EP tube.

3. Place on the ice for 30min, and swirl 3 times with the maximum rotation of the vortex instrument, 10s each time, to ensure that the cells or tissues are fully cracked.

4. Centrifuge at 4℃ for 12000g×15min, transfer the supernatant into a new 1.5mL EP tube, and then continue the next experiment after subassembly or store it at -80℃ for use.
