# Supplementary material for: Bawei Chenxiang Wan ameliorates right ventricular hypertrophy in rats with high altitude heart disease by SIRT3-HIF1α-PDK/PDH signaling pathway improving fatty acid and glucose metabolism
Source: BMC Complement Med Ther. 2024 May 15;24:190. doi: 10.1186/s12906-024-04490-6 (PMC11094862; doi:10.1186/s12906-024-04490-6)
Supplement: Supplementary file 2 — Supplementary Material 2. [file 12906_2024_4490_MOESM2_ESM.pdf]

Supplementary materials

Figure S1

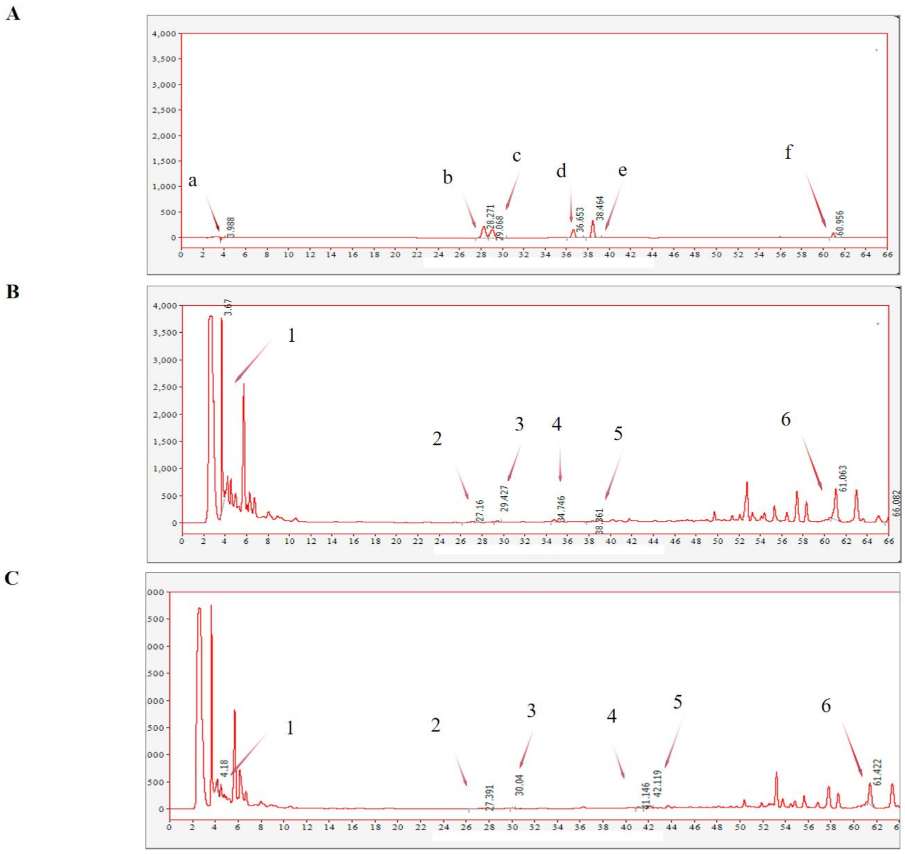

Figure 1: High-performance liquid chromatography (HPLC)

Chromatogram of the BCW extract. A representative HPLC chromatogram from three batches is shown. (A) HPLC chromatograms of mixed reference solutions of six standards: (a) β-Sitosterol, (b) luteolin, (c) Kaempferol, (d) Costunolid, (e) Naringenin and (f) Quercetin. (B) Six main chemical markers were identified in the BCW methanol extract. The denotation peaks 1–6were (1) β-Sitosterol, (2) luteolin, (3)

Kaempferol, (4) Costunolid, (5) Naringenin and (6) Quercetin.(C) Six main chemical markers were identified in the BCW water extract. The denotation peaks 1–6were (1) β-Sitosterol, (2) luteolin, (3) Kaempferol, (4) Costunolid, (5) Naringenin and (6) Quercetin.

Figure S2

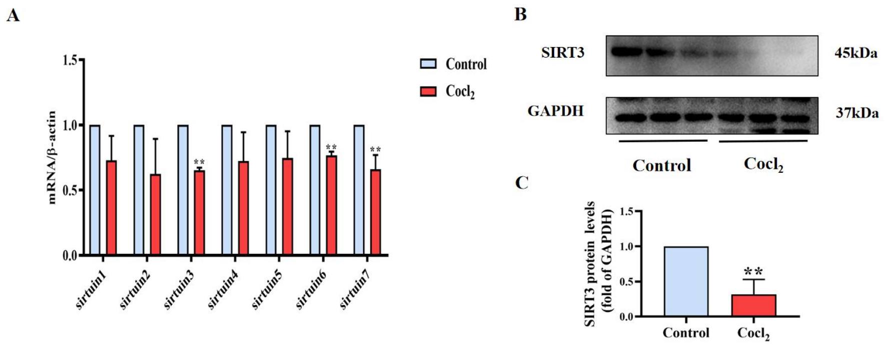

Figure S2: Changes in SIRT3 mRNA and protein expression in the $CoCl_2$-induced cell hypoxia model

(A) Real-time quantitative polymerase chain reaction analysis of SIRT1-7 mRNA levels in vitro. GADPH was used to normalize mRNA expression. (B) The protein expression of SIRT1-7 in vitro was detected by Western blot. Densitometric analysis of (C) immunoblots is presented as the ratio of the control. Results are expressed as mean ±SE: $^{*}p<0.05$, $^{**}p<0.01$vs Control, $n=3$.

Figure S3

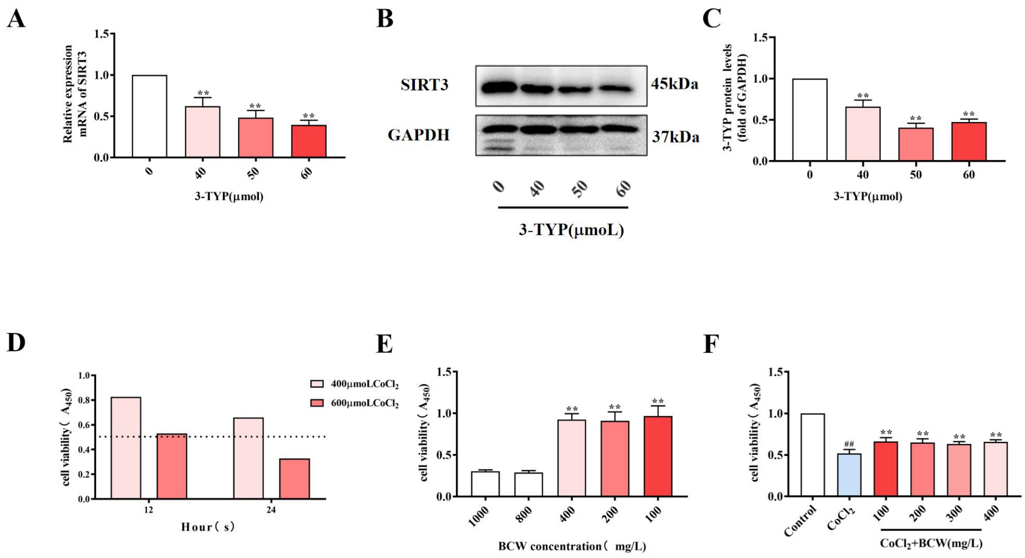

Figure S3: Screening of 3-TYP(an inhibitor of SIRT3),CoCl2 and BCW effective concentration and action time

(A) Real-time quantitative polymerase chain reaction analysis of 3-TYP mRNA level expression, and mRNA expression was normalized by GADPH. (B) The effective inhibitory concentration of 3-TYP was screened by immunoblotting. (C) Densitometric analysis of immunoblotting is expressed as the ratio of control. (D) Election of the optimal time and concentration of $CoCl_2$. (E) Safety range screening of BCW. (F) The protective effect of BCW on $CoCl_2$-induced hypoxic cell model was detected by cell viability, *n*=3.

Table S1：Compounds from BCW

	β-Sitosterol	luteolin	Kaempferol	Costunolid	Naringenin	Quercetin
Retentiontime(min)	3.670	27.160	29.427	34.746	38.361	61.063
Content(mg/g)	38.90 ± 0.01	0.13 ± 0.01	0.09 ± 0.01	0.24 ± 0.01	0.04 ± 0.01	7.23 ± 0.01

Six compounds (β-Sitosterol, luteolin, Kaempferol, Costunolid, Naringenin, Quercetin) were identified from BCW, and the minimum amount in mg/g of extract,n=3.
