# Supplementary material for: Bawei Chenxiang Wan ameliorates right ventricular hypertrophy in rats with high altitude heart disease by SIRT3-HIF1α-PDK/PDH signaling pathway improving fatty acid and glucose metabolism
Source: BMC Complement Med Ther. 2024 May 15;24:190. doi: 10.1186/s12906-024-04490-6 (PMC11094862; doi:10.1186/s12906-024-04490-6)

Figure 3 (A and H): Original WB of CPT1α, CS, and GAPDH in myocardial tissue of each group. (From left to right are Control group, RVH group, RVH+BCW0.8 g $kg^{-1}d^{-1}$ group , RVH+BCW0.4 g kg^{-1} d^{-1} group, RVH+TMZ group)

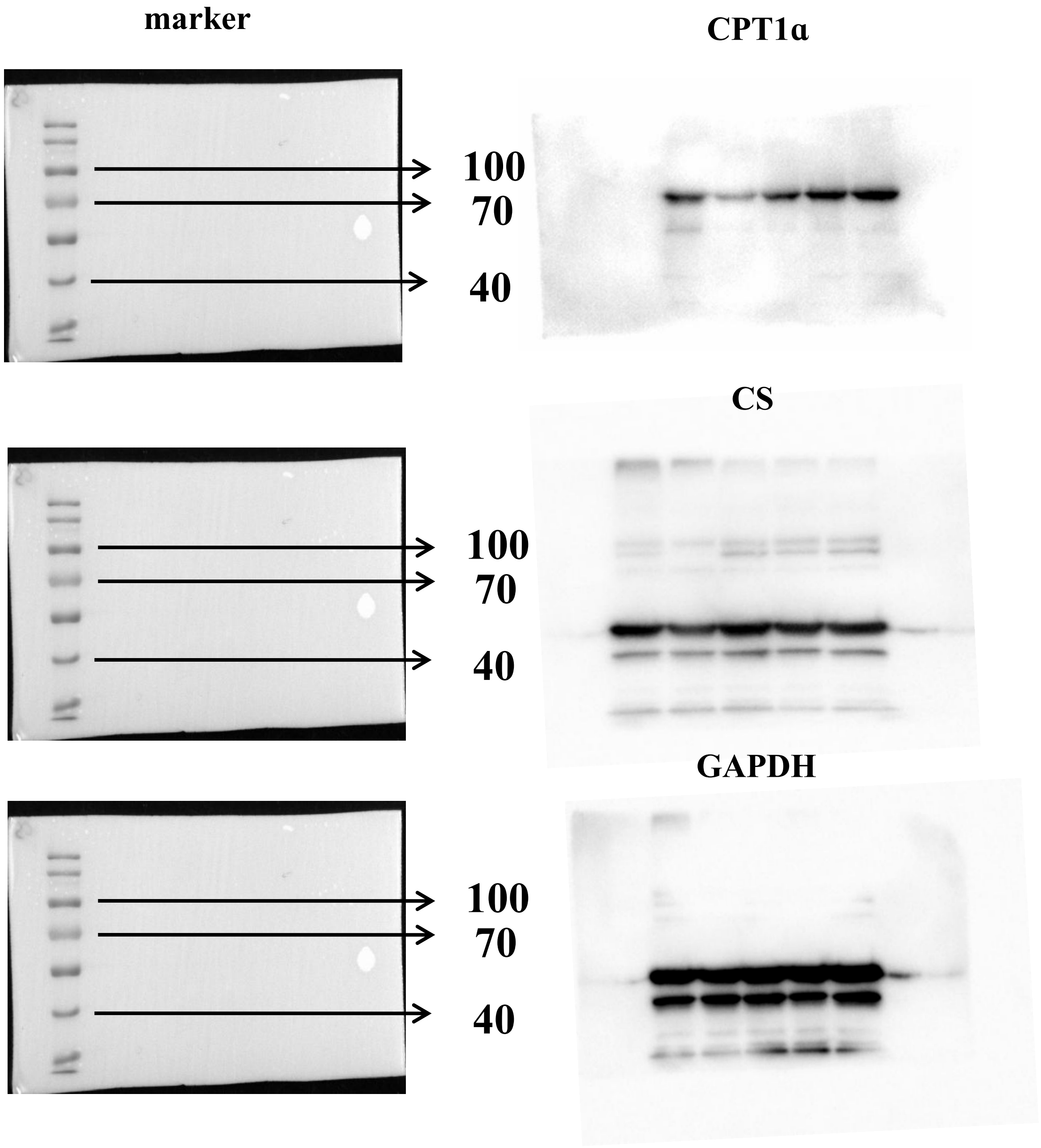

Figure 3C: Original WB of GLUT4 and GAPDH in myocardial tissue of each group. (From left to right are Control group, RVH group, RVH+BCW0.8 g $kg^{-1}d^{-1}$ group , RVH+BCW0.4 kg^{-1} d^{-1} group, RVH+TMZ group)

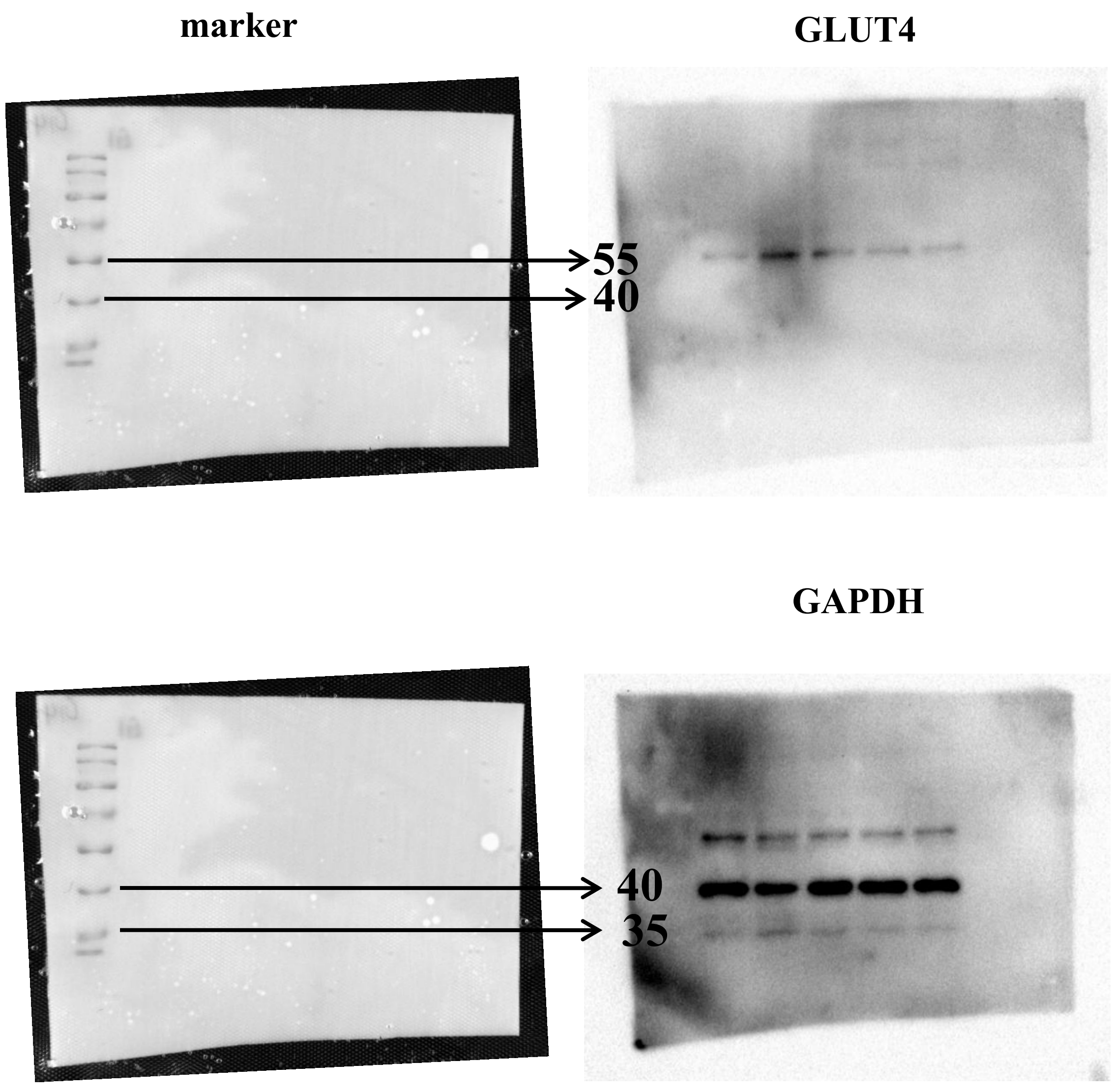

Figure 4E: Original WB of PDK1 and β-actin in myocardial tissue of each group. (From left to right are Control group, RVH group, RVH+BCW0.8 g kg^{-1}d^{-1} group , RVH+BCW0.4 kg^{-1} d^{-1} group, RVH+TMZ group)

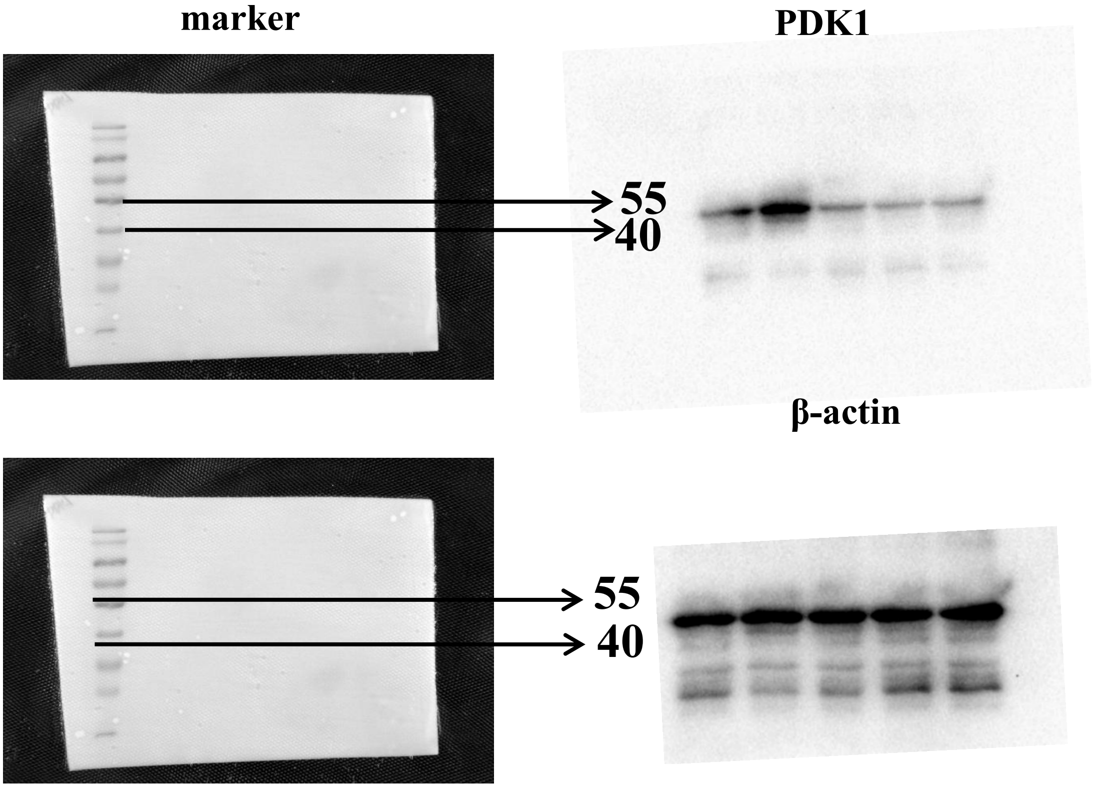

Figure 4F: Original WB of PDK2 and β-actin in myocardial tissue of each group. (From left to right are Control group, RVH group, RVH+BCW0.8 g $kg^{-1}d^{-1}$ group , RVH+BCW0.4 kg^{-1} d^{-1} group, RVH+TMZ group)

marker **PDK2**

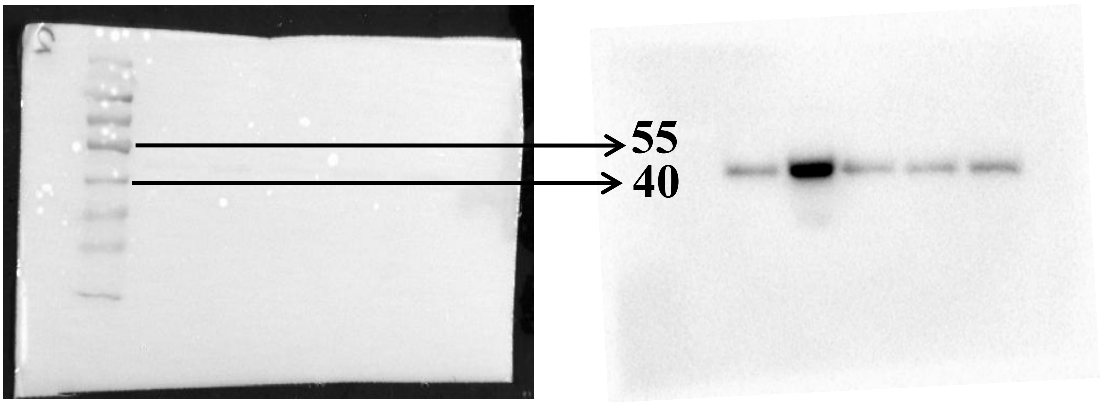

β-actin

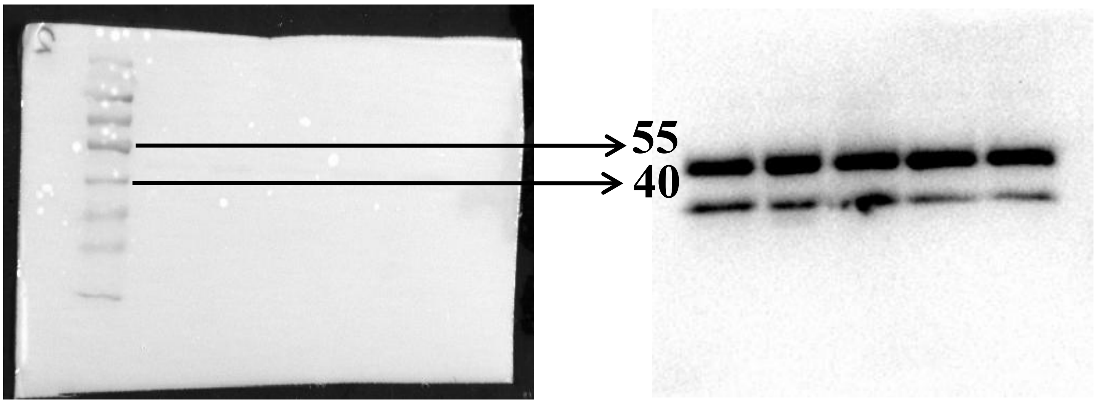

Figure 4G: Original WB of PDK2 and β-actin in myocardial tissue of each group. (From left to right are Control group, RVH group, RVH+BCW0.8 g $kg^{-1}d^{-1}$ group , RVH+BCW0.4 kg^{-1} d^{-1} group, RVH+TMZ group)

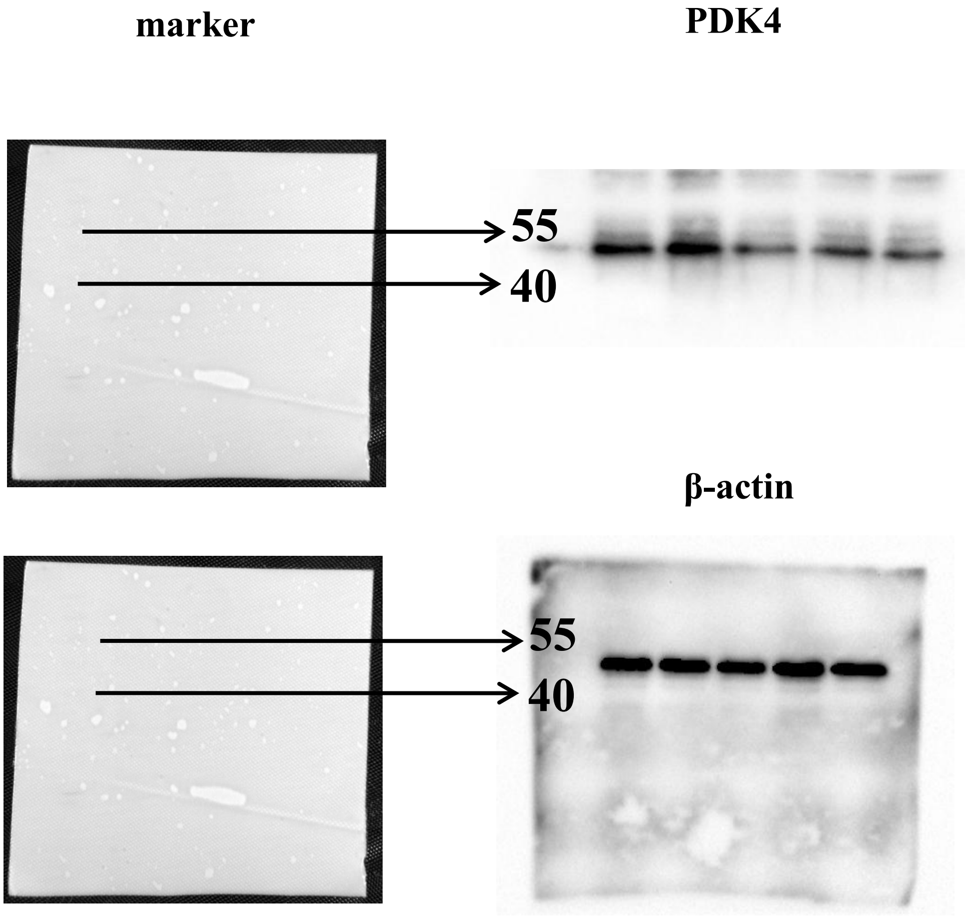

Figure 5B Original WB of p-PDH,PDH and α-Tubulin in myocardial tissue of each group. (From left to right are Control group, RVH group, RVH+BCW0.8 g $kg^{-1}d^{-1}$ group , RVH+BCW0.4 kg^{-1} d^{-1} group, RVH+TMZ group)

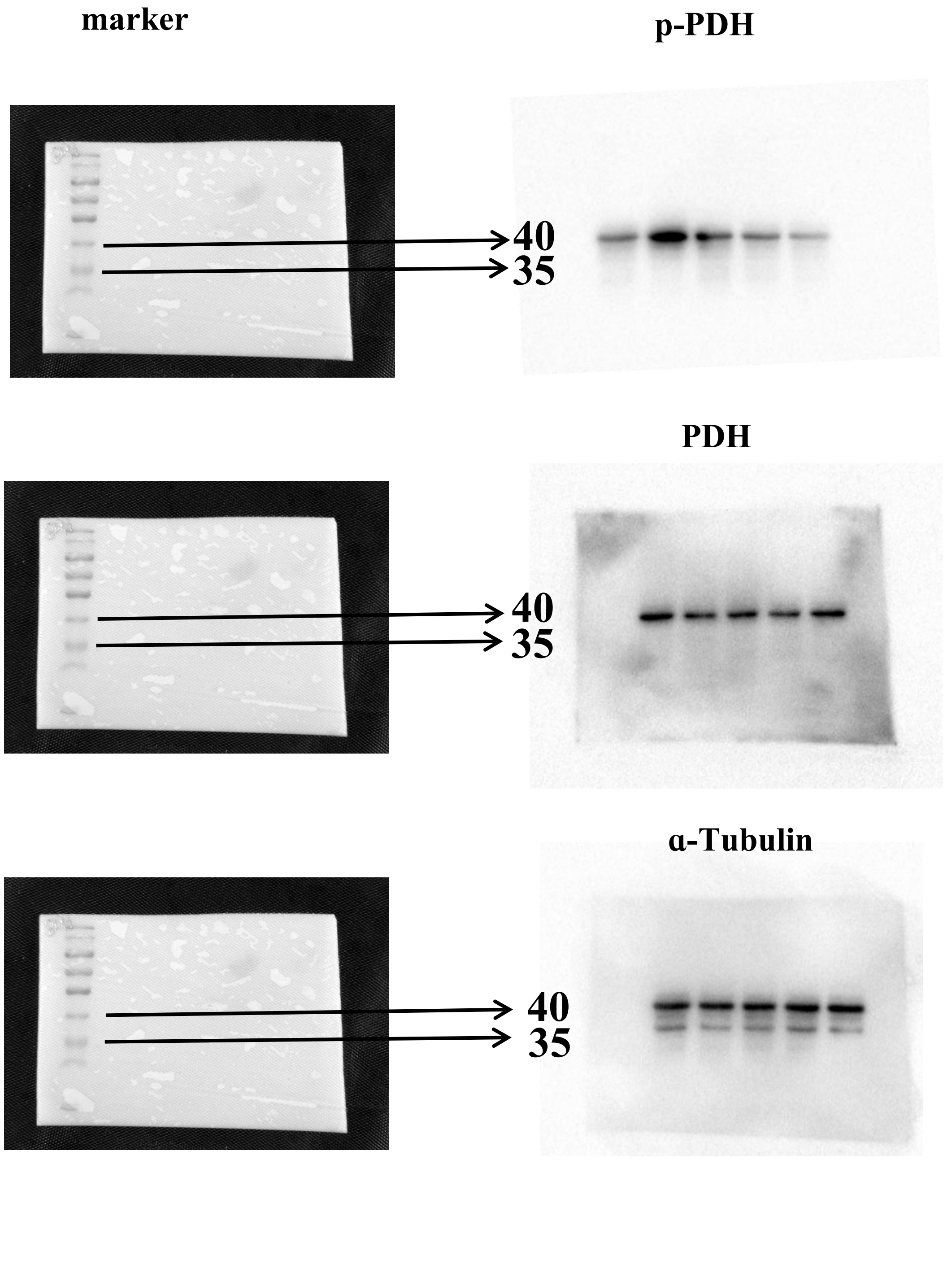

Figure 6B: Original WB of SIRT3 and GAPDH in myocardial tissue of each group. (From left to right are Control group and RVH group)

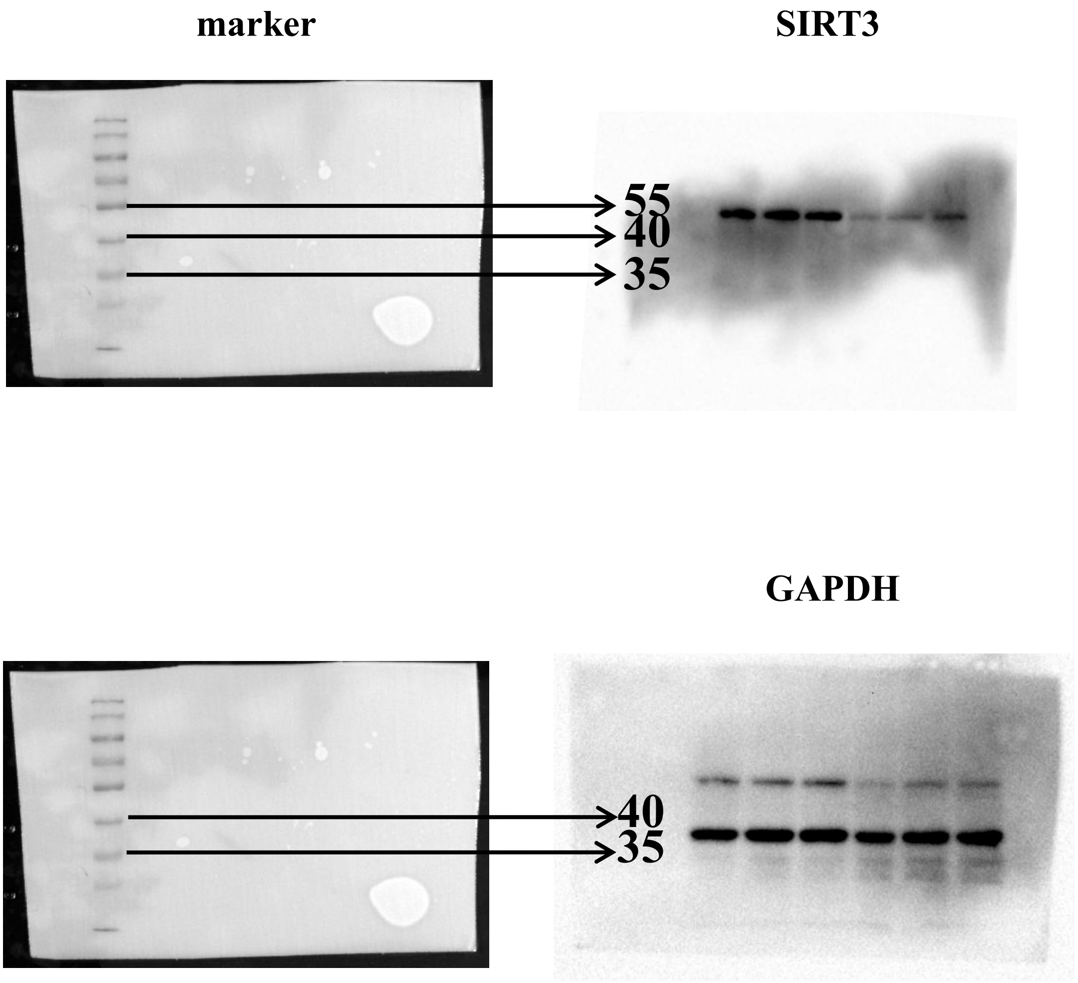

Figure 6D: Original WB of SIRT3 and GAPDH in myocardial tissue of each group. (From left to right are Control group, RVH group, RVH+BCW0.8 g $kg^{-1}d^{-1}$ group , RVH+0.4 g BCW kg^{-1} d^{-1} group, RVH+TMZ group)

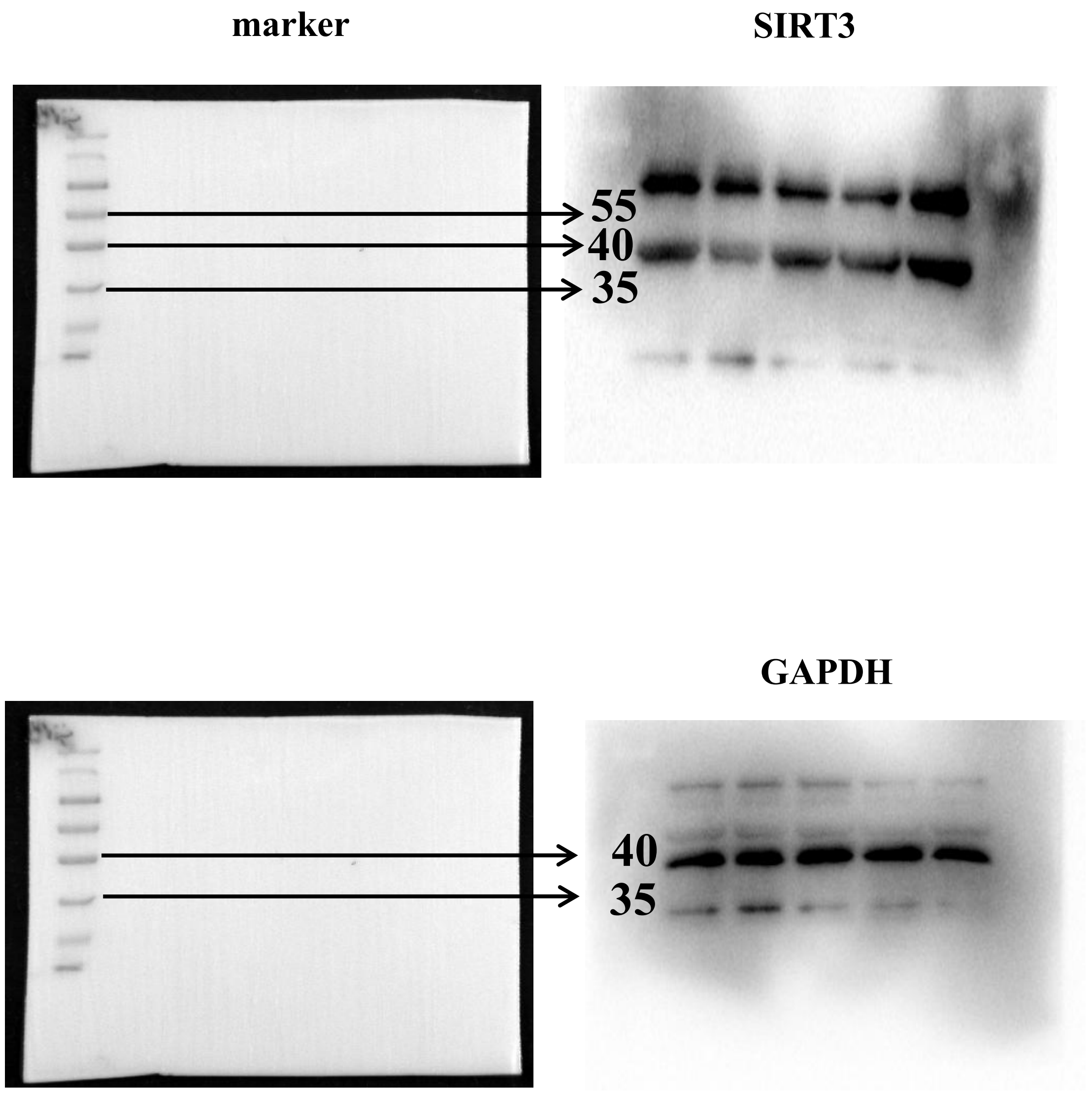

Figure 6F: Original WB of HIF1α and GAPDH in myocardial tissue of each group. (From left to right are Control group, RVH group, RVH+BCW0.8 g $kg^{-1}d^{-1}$ group , RVH+0.4 g BCW kg^{-1} d^{-1} group, RVH+TMZ group)

HIF1α

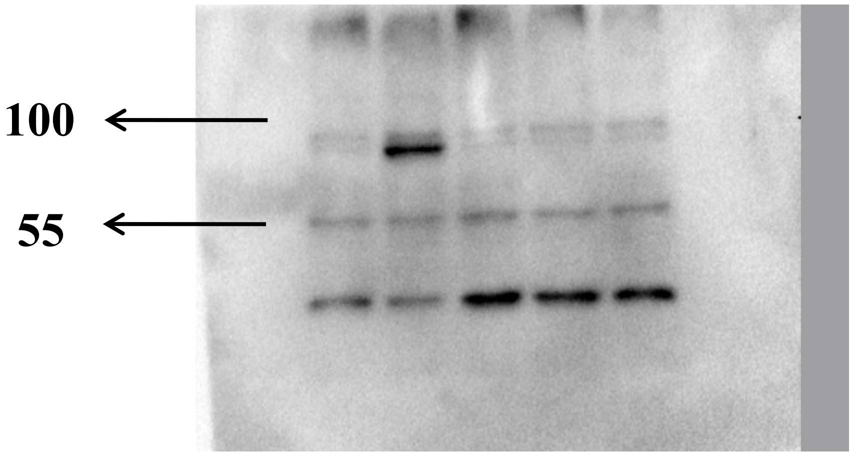

GAPDH

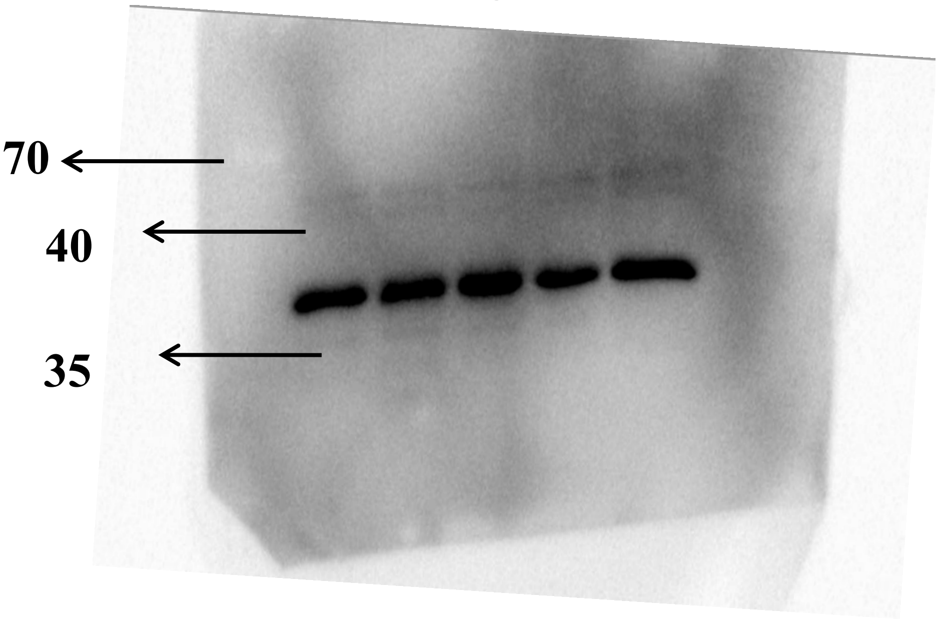

Figure 8 (A,B,C): Original WB of SIRT3, HIF1α, PDK, p-PDH, PDH and GAPDH in H9$_{C}$2 cells of each group. (From left to right are Control group, $CoCl_2$ group, $CoCl_2$+BCW group , $CoCl_2$+BCW+3TYP group)

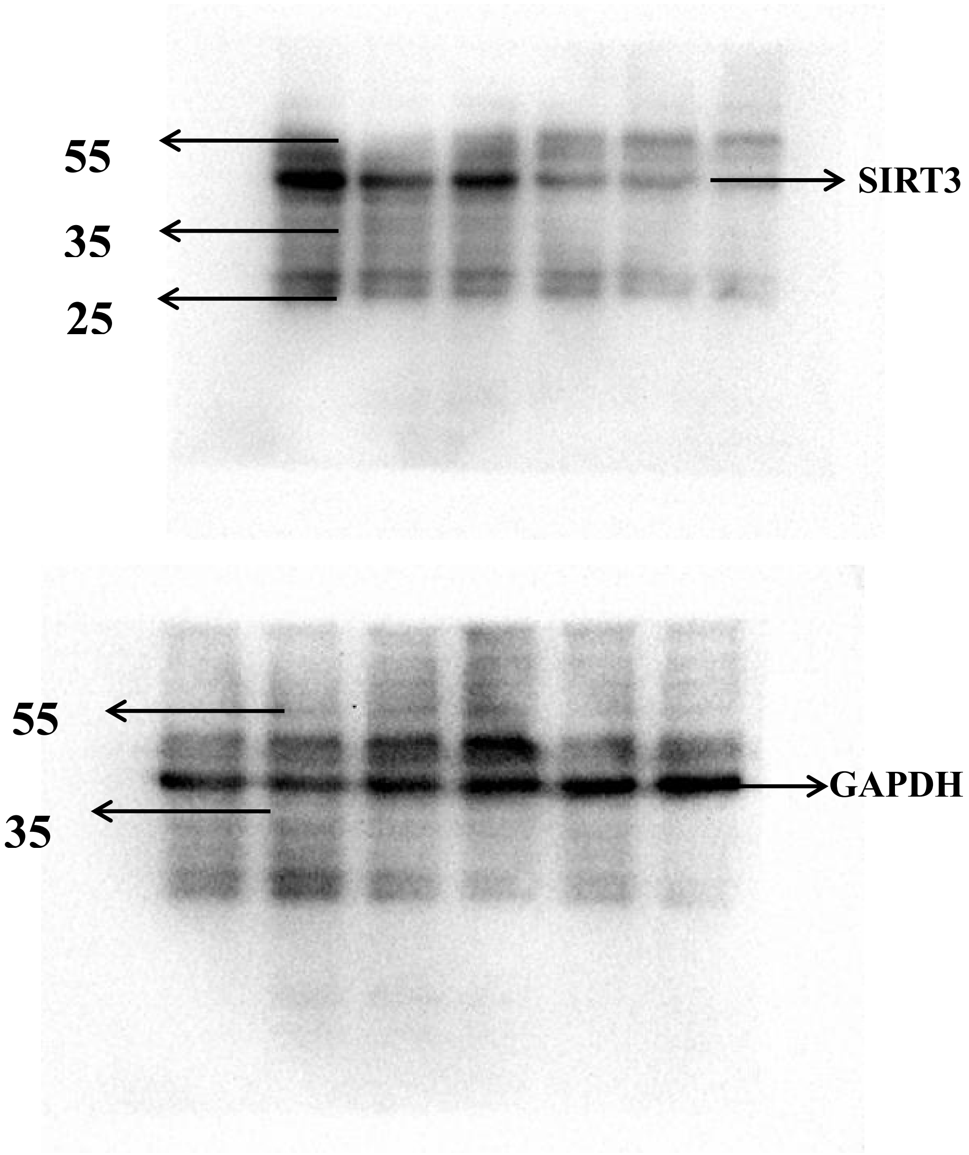

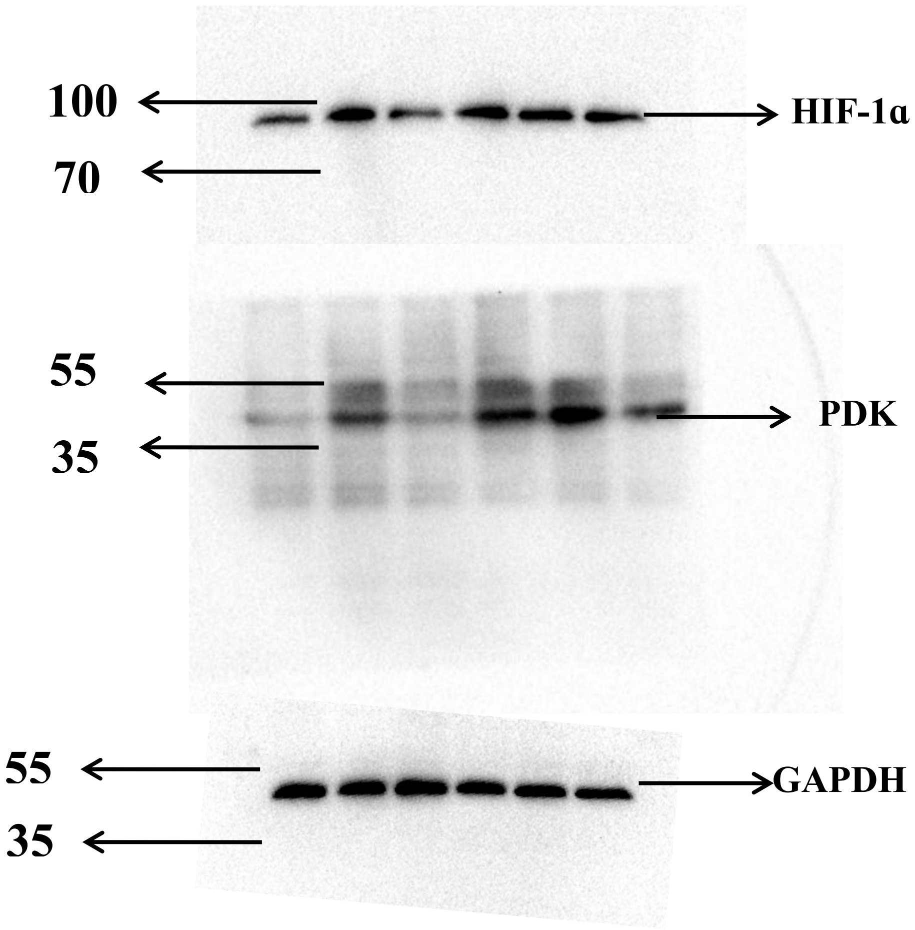
100
70
HIF-1α
55
35
PDK
55
35
GAPDH

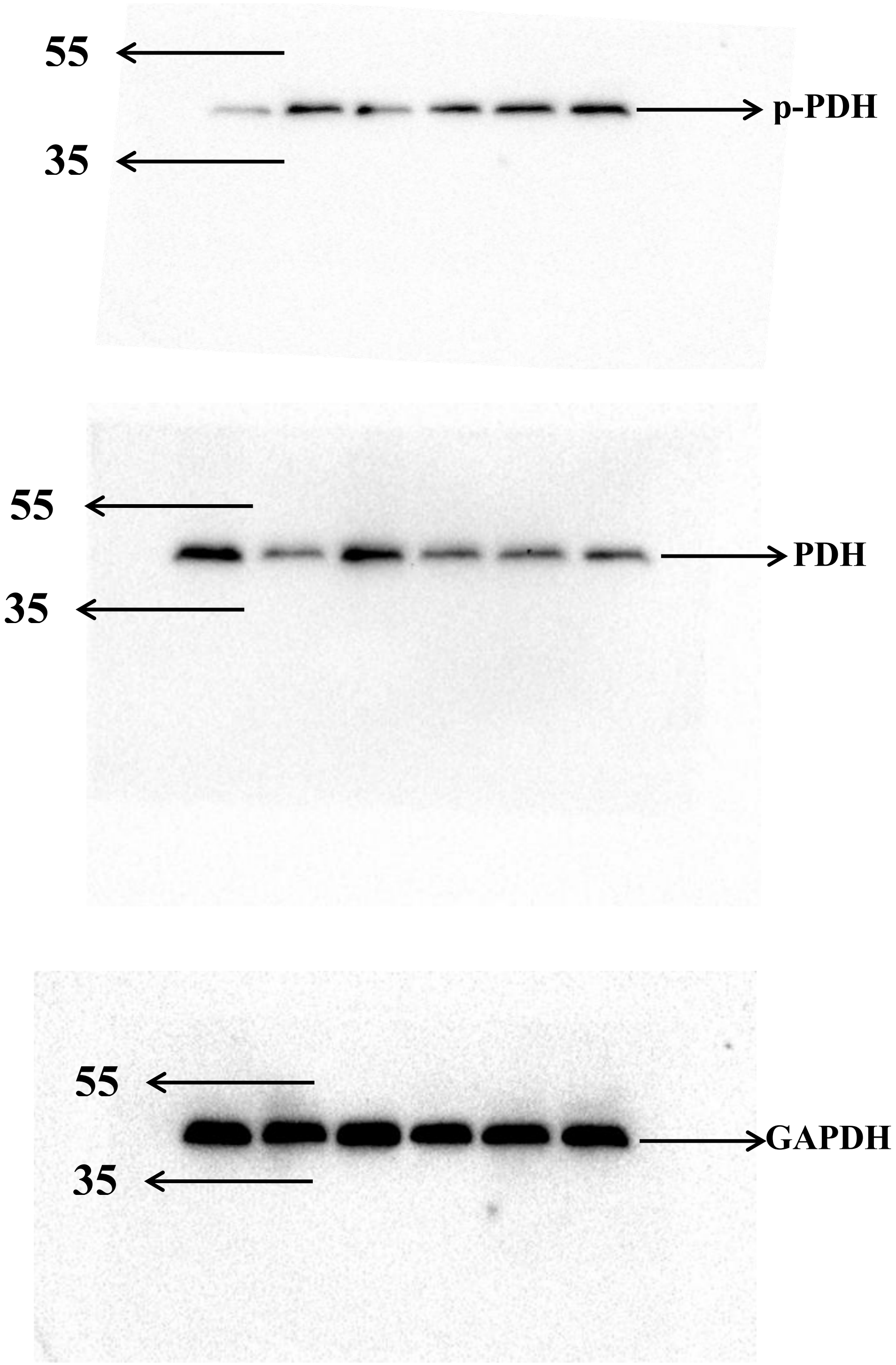
55
35
p-PDH
55
35
PDH
55
35
GAPDH

Figure S2B: Original WB of SIRT3 and GAPDH in H9C2 cells of each group. (From left to right are Control group and $CoCl_2$ group)

marker **SIRT3**

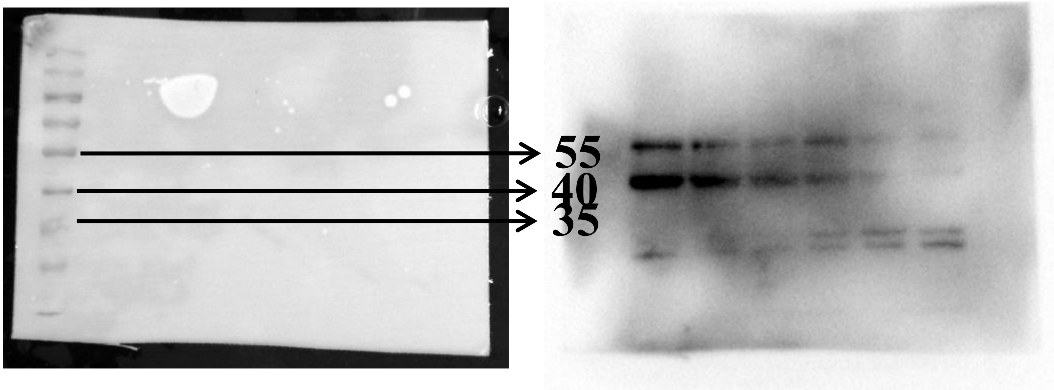

GAPDH

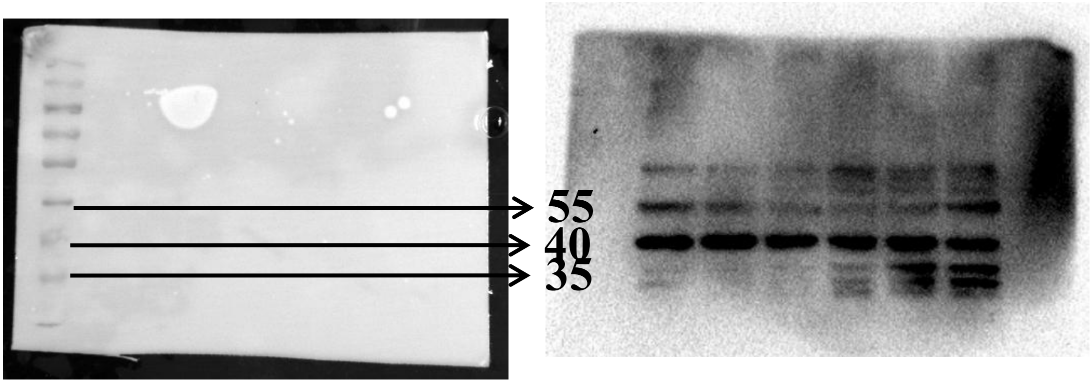

Figure S3B: Original WB of SIRT3 and GAPDH in H9$_{C}$2 cells of each group. (From left to right are 0μmoL 3TYP group, 40μmoL 3TYP group, 50μmoL 3TYP group group , 60μmoL 3TYP group)

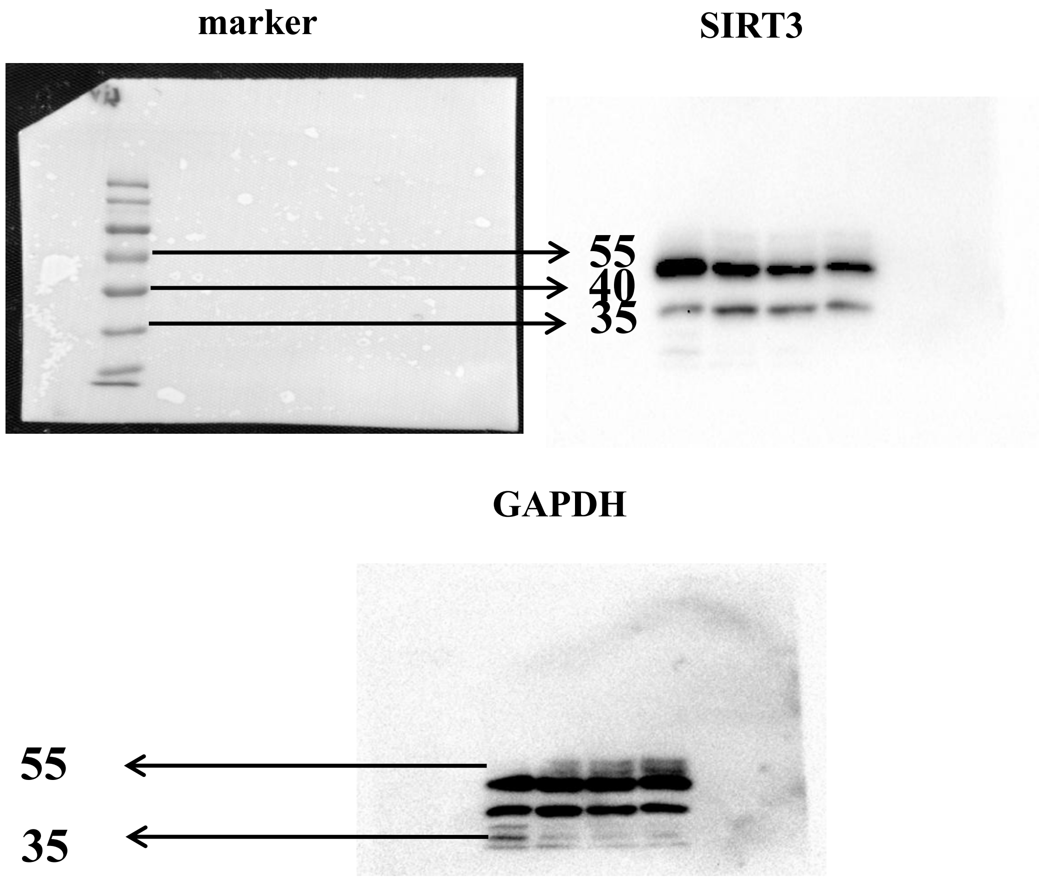

Supplement: Supplementary file 3 — Supplementary Material 3. [file 12906_2024_4490_MOESM3_ESM.pdf]
